# Acinetobacter baumannii Kills Fungi via a Type VI DNase Effector

**DOI:** 10.1128/mbio.03420-22

**Published:** 2023-01-10

**Authors:** Jingjing Luo, Xiao Chu, Jing Jie, Yu Sun, Qingtian Guan, Dan Li, Zhao-Qing Luo, Lei Song

**Affiliations:** a Department of Respiratory Medicine, Center for Infectious Diseases and Pathogen Biology, Key Laboratory of Organ Regeneration and Transplantation of the Ministry of Education, State Key Laboratory for Zoonotic Diseases, The First Hospital of Jilin University, Changchun, China; b The First Hospital of Jilin University, Changchun, China; c Department of Biological Sciences, Purdue University, West Lafayette, Indiana, USA; University of Washington

**Keywords:** type VI secretion system, anti-fungal effector, interkingdom competition, polymicrobial niches

## Abstract

Many Gram-negative bacteria deploy a type VI secretion system (T6SS) to inject toxins into target cells to promote their survival and replication in complex environments. Here, we report that Acinetobacter baumannii uses its T6SS to kill fungi and that the effector TafE (*ACX60_15365*) is responsible for such killing. Although ectopically expressed TafE is toxic to both Escherichia coli and Saccharomyces cerevisiae, deletion of *tafE* only affects the antifungal activity of A. baumannii. We demonstrate that TafE is a DNase capable of targeting the nuclei of yeast cells and that an Ntox15 domain is essential for its ability to degrade DNA. Furthermore, our findings show that A. baumannii is protected from the toxicity of TafE by elaborating the immunity protein TaeI (*ACX60_15360*), which antagonizes the activity of the effector by direct binding. The discovery of A. baumannii T6SS effectors capable of killing multiple taxonomically distinct microbes has shed light on a mechanism of the high-level fitness of this pathogen in environments characterized by scarce nutrients and the potential presence of diverse microorganisms.

## INTRODUCTION

Bacteria have evolved various mechanisms to survive in their ecological niches by establishing mutualistic and/or competitive relationships with other microbes living in the same environment. For example, commensal interactions have been documented in which Acinetobacter sp. strain C6 enables the partner organism Pseudomonas putida to grow on benzyl alcohol as a sole carbon source (1, 2). In many other scenarios, the relationship among microbes in a given niche is competition for space and resources. A number of antagonistic strategies used by bacteria have been described, including effective assimilation of key nutrients (e.g., sequester of iron by siderophores), modification of the microenvironmental conditions to make them less suitable for other bacteria to live, and the production of antimicrobials that eliminate or slow the growth of proximal microbes (3).

The type VI secretion system (T6SS) is a widely distributed nanomachine employed by many bacteria to kill competitors in polymicrobial communities (4). The antimicrobial activity of these machines is mediated by toxic effectors injected into prey cells (5, 6). Although most characterized T6SS effectors are weapons elaborated to target Gram-negative bacteria (7), some have been shown to promote virulence by directly interfering with host cellular processes (8–10) or by suppressing immunity (11). In addition, effectors that facilitate bacterial growth by assimilating scarce metal ions have also been reported (12, 13).

The cell wall of Gram-positive bacteria was previously believed to be impenetrable by T6SS spears due to its thick peptidoglycan layers and the presence of teichoic acid polymers. The plant pathogen Acidovorax citrulli utilizes its T6SS to kill both Gram-positive bacteria and fungi (14), which have a cell wall that is made of polymers of mannose, glucose, and *N*-acetylglucosamine and is thicker than that of Gram-positive bacteria (15). Antifungal activity has also been documented for Serratia marcescens (16), Klebsiella pneumoniae (17), and Myxococcus xanthus (18).

Acinetobacter baumannii is a Gram-negative aerobic short bacillus that poses grave challenges in hospital environments as an important nosocomial pathogen. As one of the “ESKAPE” pathogens (Enterococcus faecium, Staphylococcus aureus, K. pneumoniae, A. baumannii, Pseudomonas aeruginosa, and Enterobacter species) (19), A. baumannii is notorious for its ability to cause deadly hospital-acquired infections, which are difficult or even impossible to treat due to its extensive acquisition of resistance against multiple antibiotics (20). A. baumannii is also known for its ability to survive for long durations on dry surfaces, such as medical equipment, and in niches of scarce nutrients in a range of temperatures and pH conditions (21, 22). These features suggest that this bacterium has evolved mechanisms to effectively assimilate nutrients from the environment and to outcompete other microbes. Indeed, A. baumannii is equipped with mechanisms to effectively acquire nutrients, such as essential metals, from the environment, including its hosts (23).

Bioinformatics analysis has revealed that A. baumannii codes for a T6SS and a large cohort of putative effectors that are conserved among different isolates (24–26). In strain 17978 (27) and some recently isolated multidrug resistance strains, the expression of the T6SS is repressed by two TetR-like proteins harbored on the large plasmid pAB3, suggesting coordination between plasmid conjugation and the activation of the competitor-killing machine (28). A recent study found that the T6SS of A. baumannii mediates killing of Gram-positive bacteria by a unique mechanism involving the secretion of d-lysine, which modulates the extracellular pH to potentiate the peptidoglycanase activity of the effector Tse4 (29).

Here, we report that A. baumannii kills fungi via its T6SS effector TafE (*ACX60_15365*), which is a DNase harboring nuclear localization signals for yeast cells. We also demonstrate that the toxicity of TafE against A. baumannii is prevented by the immunity protein TaeI (*ACX60_15360*), which blocks its enzymatic activity by direct protein-protein interactions.

## RESULTS

### Derepression of T6SS enables A. baumannii to kill bacteria and fungi.

**FIG 1:**
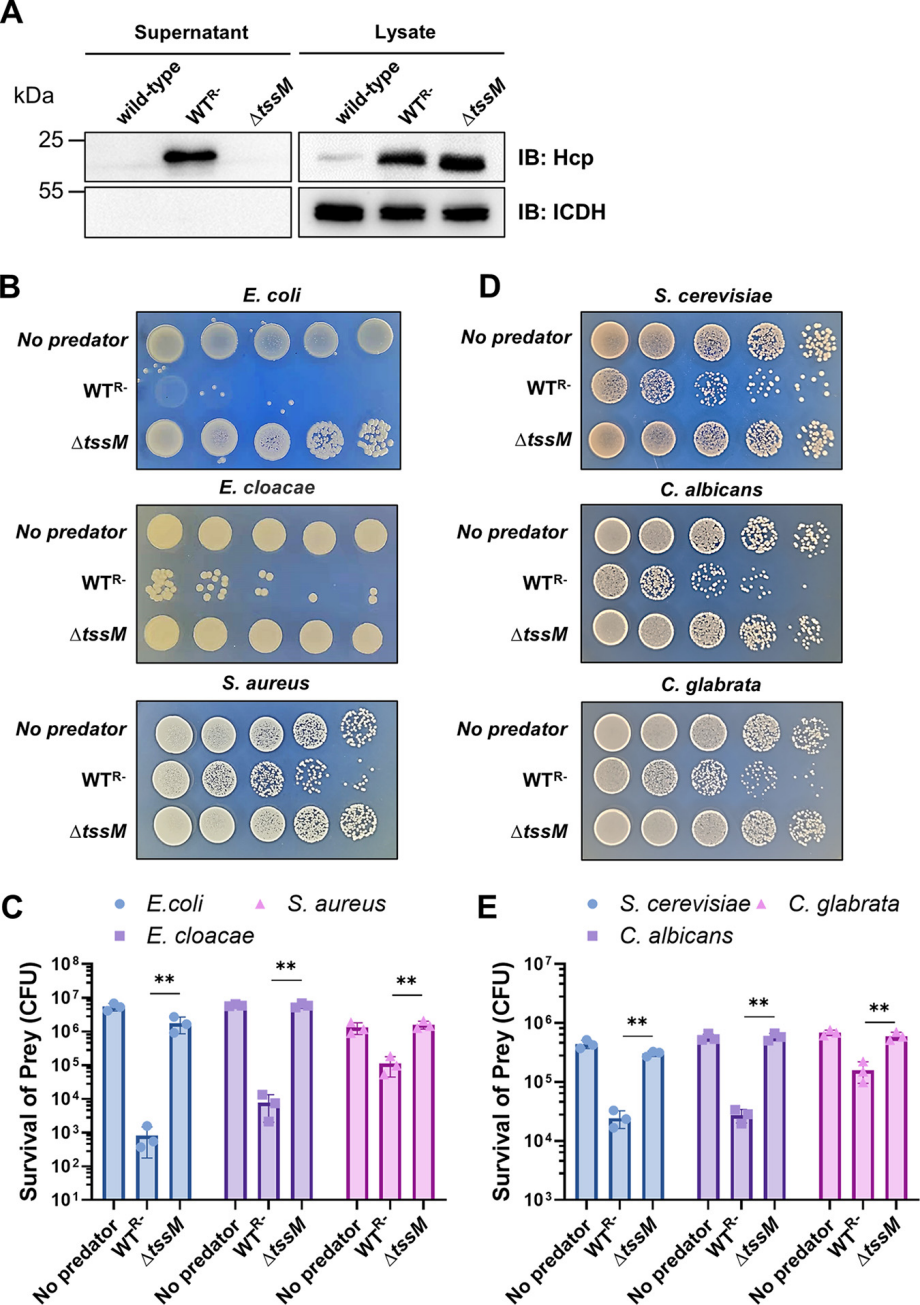
Curing of the pAB3 plasmid in A. baumannii strain 17978 derepresses its T6SS to kill bacteria and fungi. (A) The pAB3 plasmid represses the expression of T6SS in strain 17978. Culture supernatant of the indicated bacterial strains was analyzed for secreted Hcp, one component of T6SS’s puncturing structure. Note that the strain lacking pAB3 secretes Hcp and deletion of *tssM* abolished such secretion. The cytosolic isocitrate dehydrogenase (ICDH) was used as an internal reference to evaluate the integrity of bacterial cells. (B, C) T6SS-dependent killing of Gram-negative and Gram-positive bacteria. (B) Cells of the wild-type A. baumannii or the Δ*tssM* mutant were mixed with the indicated bacteria at a 1:1 ratio for 4 h. The survival of the prey bacteria was assessed by plating dilutions on selective medium. (C) The survival rates were calculated by dividing the counts of surviving bacteria by the input. (D, E) T6SS-dependent killing of three yeast species. (D) Cells of the indicated A. baumannii strains and yeast were mixed at a 10:1 ratio for 4 h, and killing was evaluated by plating dilutions of the cells on selective medium. (E) Quantitation was performed by determining the number of surviving yeast cells and dividing by the input. The results in panels C and E were from three independent experiments each done in triplicate. Error bars show standard deviations. **, *P < *0.01.

A. baumannii strain 17978 has been shown to carry a large plasmid called pAB3, which harbors two TetR-like regulatory proteins that inhibit the expression of its T6SS genes (28). Consistent with this notion, the wild-type strain harboring pAB3 (designated the wild type [WT]) did not detectably express or secrete the hemolysin-coregulated protein (Hcp) (Fig. 1A). In contrast, curing of pAB3 generated a strain (designated WT^R−^) (Table S1 in the supplemental material) that robustly expressed and secreted Hcp into the culture supernatant (Fig. 1A). To further determine the role of the T6SS in the secretion of Hcp, we created a mutant lacking *tssM* (Table S1), which codes for an essential component of the T6SS belonging to the IcmF (intracellular multiplication protein F) protein family (30, 31). As expected, the Δ*tssM* mutant completely lost the ability to secrete Hcp (Fig. 1A).

10.1128/mbio.03420-22.8 TABLE S1 Bacterial strains, plasmids, and primers used in this study. Download Table S1, DOCX file, 0.02 MB. Copyright © 2023 Luo et al. 2023 Luo et al. https://creativecommons.org/licenses/by/4.0/ This content is distributed under the terms of the Creative Commons Attribution 4.0 International license.

The T6SS of A. baumannii strain 17978 functions to kill nonkin bacteria in polymicrobial niches (28–30). We thus examined the ability of the WT^R−^ strain to kill Escherichia coli and Enterobacter cloacae, two Gram-negative bacteria, as well as the Gram-positive bacterium S. aureus. Coincubation of the WT^R−^ strain with E. coli or E. cloacae led to significant reduction of viable cells of both bacteria, and such killing did not occur when the Δ*tssM* mutant was used in similar experiments (Fig. 1B and C). Interestingly, although at a detectably lower efficiency, A. baumannii also killed S. aureus (Fig. 1B and C). The lower killing efficiency against Gram-positive bacteria is consistent with the fact that this group of microorganisms are more recalcitrant to T6SS-mediated killing, due at least in part to their thicker cell wall.

Because of their powerful metabolic capacity in degrading polymers not consumable by most bacteria, fungi often are the first colonizers in niches originally less suitable for prokaryotes (32). Byproducts of fungal metabolism then attract bacteria to form polymicrobial communities where the latter may need to outcompete the eukaryote in order to succeed in establishing its niche (33). A. baumannii has the ability to survive in nutrient-scarce environments, such as the surface of medical instruments. We therefore examined whether it could kill fungal cells. Relevant strains of A. baumannii were mixed with the budding yeast Saccharomyces cerevisiae, as well as Candida albicans and Candida glabrata, two fungal pathogens frequently found in nosocomial environments. The A. baumannii strain WT^R−^ with an active T6SS killed each of these three fungal species to varying degrees. Again, deletion of *tssM* abolished its ability to compete against fungal cells (Fig. 1D and E). These results indicate that A. baumannii utilizes its T6SS to attack a wide range of microbial competitors, including bacteria and fungi.

### The T6SS effector TafE is required for the antifungal activity of A. baumannii.

The valine glycine repeat G (VgrG) proteins are a family of secreted T6SS components that are essential in the assembly of the nanomachine’s baseplate (34, 35). Another important role of VgrGs is their involvement in the delivery of cognate effectors encoded by proximal genes (36). These proteins also exert effector function through activity conferred by their carboxyl domains (35, 37, 38). Bioinformatics analysis revealed that A. baumannii strain 17978 codes for four VgrG clusters, each linked to a putative effector encoded by an immediately downstream gene. These putative effectors are Tle (*ACX60_17660*), TafE (*ACX60_15365*), Tse (*ACX60_11695*), and Tae (*ACX60_00605*) (Fig. S1A). We first determined the toxicity of these effectors to E. coli by introducing plasmids expressing each of them into strain BL21(DE3). Induction of protein expression by IPTG (isopropyl β-d-thiogalactopyranoside) arrested the growth of strains expressing Tle, TafE, or Tae (Fig. S1B and C). Intriguingly, although its expression was readily detectable, Tse did not display discernable toxicity to E. coli (Fig. S1C), suggesting that this effector has only a very subtle impact on the bacterium or it plays another role in the biology of A. baumannii (Fig. S1B).

10.1128/mbio.03420-22.1 FIG S1 Putative T6SS effectors of Acinetobacter baumannii strain 17978. (A) Cluster and gene organization of putative T6SS effectors. Predicted *vgrG* genes and their putative cognate effector genes are depicted as orange and grey arrows, respectively. The potential genes for the immunity protein downstream from their effectors are represented by light blue arrows. (B) Toxicity of putative T6SS effectors to Escherichia coli. Putative effector genes were cloned into pET-sumo; each of the resulting plasmids was introduced into E. coli strain BL21(DE3), and toxicity was determined by spotting dilutions of bacterial cells onto LB plates with and without IPTG. Growth was assessed by acquiring the images after incubation at 37°C for 14 h for LB plates without IPTG or at 25°C for 2 days for LB plates with IPTG. (C) Expression of putative T6SS effectors in E. coli. The expression of the putative effectors was determined after IPTG induction by immunoblotting with anti-His_6_ antibodies. Download FIG S1, TIF file, 2 MB. Copyright © 2023 Luo et al. 2023 Luo et al. https://creativecommons.org/licenses/by/4.0/ This content is distributed under the terms of the Creative Commons Attribution 4.0 International license.

**FIG 2:**
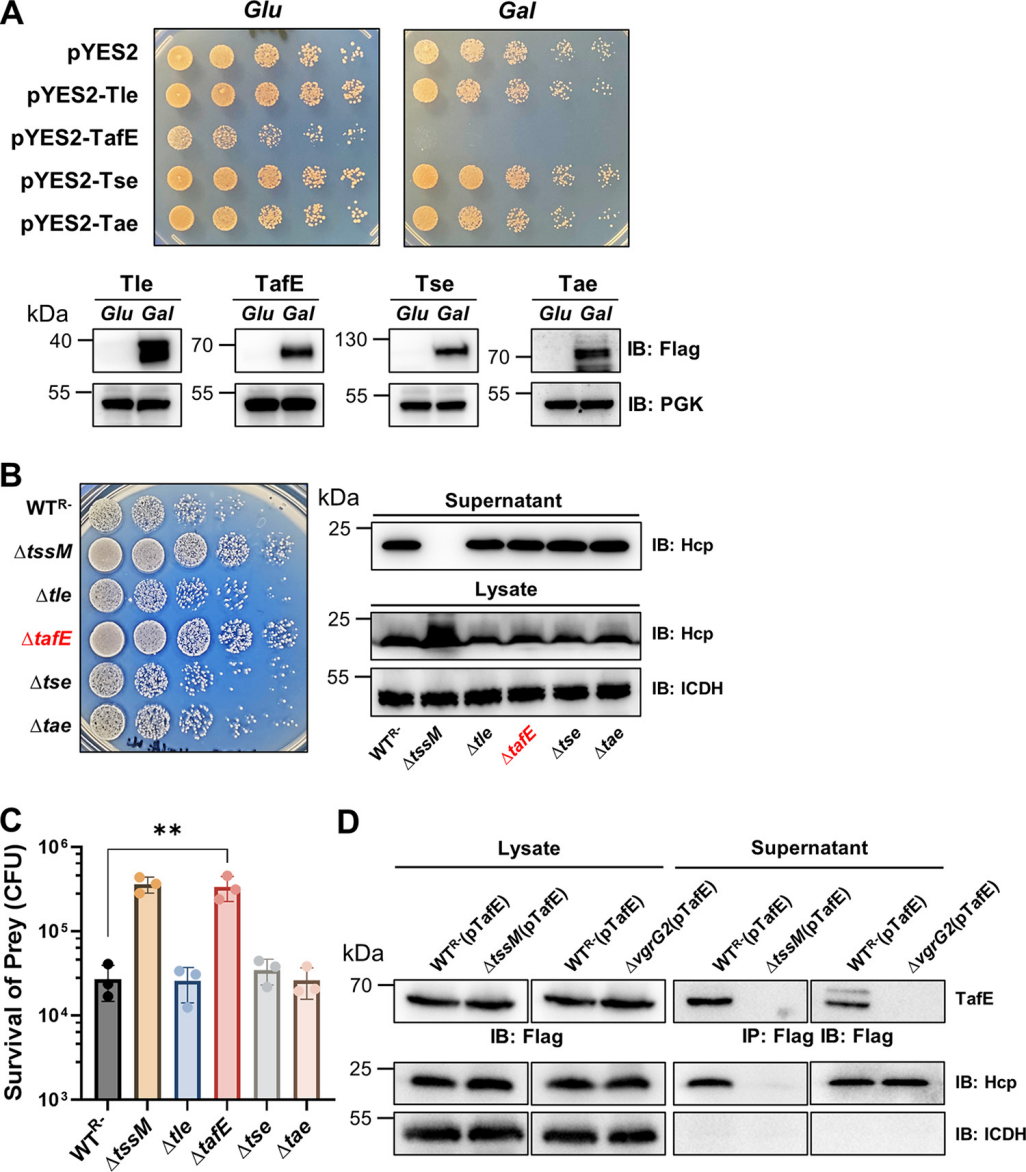
Yeast killing by A. baumannii requires the effector TafE. (A) Identification of TafE as a yeast-toxic protein. A few predicted T6SS effectors from A. baumannii were tested for yeast toxicity. Top, cells of yeast strains harboring plasmids carrying the Flag-tagged testing genes expressed from the P_gal_ promoter were spotted onto synthetic medium containing 2% glucose or 2% galactose. Images were acquired after incubation at 30°C for 4 days. Bottom, the expression of the proteins was detected by immunoblotting with Flag-specific antibody. The metabolic enzyme phosphoglycerate kinase (PGK) was probed as a loading control. (B, C) The *tafE* gene is essential for A. baumannii to kill yeast. (B) Left, the indicated A. baumannii strains were used to kill yeast as described in the legend to Fig. 1D. Right, the functionality of the T6SS in these strains was determined by their ability to secrete Hcp. Note that deletion of *tafE* and other putative effectors did not affect the function of the T6SS machine. (C) The survival rates were calculated by dividing the counts of surviving bacteria by the input. The results were from three independent experiments each done in triplicate. Error bars show standard deviations. **, *P* < 0.01. (D) Secretion of TafE by A. baumannii strains. A plasmid expressing Flag-TafE was introduced into the indicated A. baumannii strains, and the secretion of the fusion protein was determined by immunoprecipitation of culture supernatant with the Flag-specific antibody. The expression of the protein was determined by immunoblotting of bacterial cell lysates. Note that secretion of TafE requires TssM or VgrG2. The integrity of bacterial cells was evaluated by probing the cytosolic ICDH. IB, immunoblotting; IP, immunoprecipitation.

Next, we set out to identify the effector responsible for the antifungal activity by expressing each of these four putative effectors in S. cerevisiae on a vector harboring the galactose-inducible promoter P_gal_. Growth experiments using serially diluted cells of the relevant yeast strains on medium containing glucose or galactose revealed that only TafE exhibited toxicity (Fig. 2A). In contrast, although each was expressed at high levels, Tle, Tse, and Tae did not detectably inhibit yeast growth (Fig. 2A).

To determine whether TafE contributes to the antifungal activity of A. baumannii, we constructed a panel of mutants and used the killing assay to probe the effector directly responsible for the antifungal activity. Deletion of the gene coding for Tle, Tse, or Tae did not detectably impact the ability of A. baumannii to kill S. cerevisiae (Fig. 2B and C). Importantly, yeast cells that had been mixed with the Δ*tafE* mutant survived at levels comparable to the survival of those that had been mixed with the Δ*tssM* mutant, indicating that TafE is the effector that targets fungal cells (Fig. 2B and C). Each of the mutants lacking a specific effector gene still expressed and secreted Hcp at levels comparable to those in the WT^R−^ strain (Fig. 2B), indicating that the T6SS is functional in these mutants. Taken together, these results suggest that TafE is the key effector utilized by A. baumannii in fighting against fungi.

We also validated T6SS-mediated secretion of TafE by inserting the gene into pJL03, which allows the expression of Flag-tagged proteins in A. baumannii (39), and the resulting plasmid, pJL03-TafE, was introduced into the WT^R−^, Δ*tssM*, and Δ*vgrG2* strains. Secreted Flag-TafE could be readily detected in the culture supernatant of strain WT^R−^(pJL03-TafE) (Fig. 2D). Although the protein was similarly expressed, secretion of Flag-TafE by the Δ*tssM*(pJL03-TafE) or Δ*vgrG2*(pJL03-TafE) strain was not detectable. As expected, the expression and secretion of Hcp were not affected by overexpression of TafE or by *vgrG2* deletion (Fig. 2D). These results indicate that TafE is secreted by the T6SS of A. baumannii in a VgrG2-dependent manner.

### TafE is a Mg^2+^-dependent DNase.

**FIG 3:**
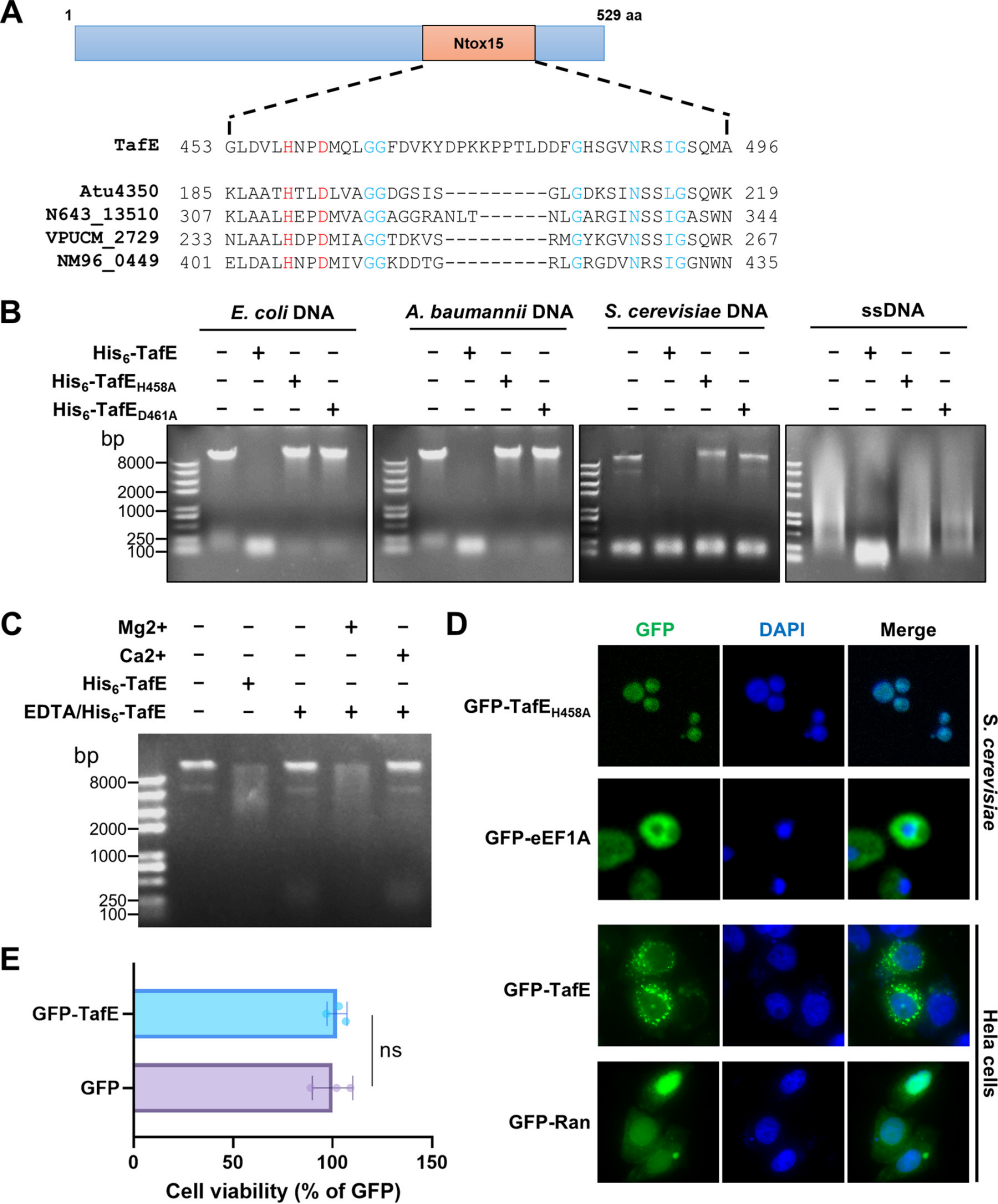
TafE is a DNase that harbors the Ntox15 domain with an HXXD motif. (A) A schematic of TafE showing the location of the predicted Ntox15 domain in the protein, with its sequence shown at the top of the alignment of the regions containing the HXXD motif among several T6SS effectors from various bacteria, including Atu4350 of Agrobacterium tumefaciens, N643_13510 of Salmonella bongori, VPUCM_2729 of Vibrio parahaemolyticus, and NM96_0449 of Neisseria mucosa. The alignment was generated using Jalview (63). aa, amino acids. (B) Degradation of DNA from different organisms by TafE. Recombinant TafE and its mutants defective in the HXXD motif were incubated with genomic DNA of E. coli, A. baumannii, or S. cerevisiae for 1 h at 37°C. A similar treatment was performed with single-stranded DNA (ssDNA) from salmon sperm. DNA degradation was assessed by agarose gel electrophoresis followed by ethidium bromide staining. (C) The DNase activity of TafE requires Mg^2+^. Recombinant TafE was treated with EDTA, and its DNase activity was determined in reaction mixtures receiving 2 mM Mg^2+^ or 2 mM Ca^2+^. Note that only Mg^2+^ restored its ability to cleave DNA after EDTA treatment. (D) TafE is targeted to the nucleus of yeast cells. Cells of S. cerevisiae expressing GFP-TafE_H458A_ or GFP-eEF1A and HeLa cells expressing GFP-TafE or GFP-Ran were fixed and stained with DAPI. Cellular localization of the fusion protein was assessed by confocal microscopy. Note that the GFP signals perfectly overlapped those of the nucleus in yeast cells. (E) TafE shows no toxicity to HeLa cells. The CCK8 assay was performed in HeLa cells expressing GFP or GFP-TafE. Note that the ectopic expression of TafE has no effects on the viability of HeLa cells. Error bars show standard deviations. ns, not significant.

TafE was annotated as a hypothetical protein, and it is predicted by the Pfam database to harbor an Ntox15 domain with an H_458_XXD_461_ (X, any amino acid) catalytic motif that is present in a wide variety of nucleases (Fig. 3A) (40). Further analysis by PSI-BLAST revealed that the carboxyl end of TafE has a high probability of having structural features similar to those found in Atu4350 from Agrobacterium fabrum (41), N643_13510 from Salmonella bongori, VPUCM_2729 from Vibrio parahaemolyticus, and NM96_04490 from Neisseria mucosa (Fig. 3A and Fig. S2).

10.1128/mbio.03420-22.2 FIG S2 Sequence alignment of the representative toxins that contain the toxin_43 domain. The alignment was generated using Jalview (63). Strictly conserved residues are shown in a dark blue background, and the HXXD motifs are marked with white letters in a red background. Proteins shown are Atu4350 of *Agrobacterium fabrum*, N643_13510 of Salmonella bongori, VPUCM_2729 of Vibrio parahaemolyticus, and NM96_0449 of Neisseria mucosa. Download FIG S2, TIF file, 2.7 MB. Copyright © 2023 Luo et al. 2023 Luo et al. https://creativecommons.org/licenses/by/4.0/ This content is distributed under the terms of the Creative Commons Attribution 4.0 International license.

To gain a more complete appreciation of the relationships of TafE within the *Pseudomonadota* phylum, we performed phylogenetic analysis of TafE proteins from the phyla *Alphaproteobacteria* and *Betaproteobacteria*, orders *Alteromonadales*, *Vibrionales*, *Pseudomonadales*, and *Moraxellales*, and several species from the Acinetobacter genus (Fig. S3). TafE proteins from each of the classes *Alphaproteobacteria*, *Betaproteobacteria*, and *Gammaproteobacteria* formed three different phyloclades. Interestingly, TafE proteins from Acinetobacter did not form a monophyletic group. Instead, TafE proteins from Acinetobacter dispersus, Acinetobacter seifertii, and Acinetobacter bereziniae together with the TafE proteins from *Pseudomonadales* formed a monophyletic group. This distribution may result from convergent evolution or from gene transfer events between the orders before the speciation of members of *Pseudomonadales*.

10.1128/mbio.03420-22.3 FIG S3 Phylogenetic tree of TafE orthologs from different bacterial species. Protein sequences of TafE orthologs from the indicated bacterial species were obtained from the Uniprot database (http://www.uniprot.org/). The phylogenetic tree was constructed by the neighbor-joining method, using MEGA 7.0. The scale bar indicates the percentage of divergence (distance). YPK_0782 of Yersinia pseudotuberculosis strain YPIII is in red, and ortholog genes of E. coli, Salmonella enterica serovar Typhimurium, S. enterica serovar Enteritidis, Corynebacterium glutamicum, and A. baumannii are in blue. Download FIG S3, TIF file, 1.8 MB. Copyright © 2023 Luo et al. 2023 Luo et al. https://creativecommons.org/licenses/by/4.0/ This content is distributed under the terms of the Creative Commons Attribution 4.0 International license.

To determine the biochemical activity of TafE, we purified E. coli His_6_-TafE, as well as His_6_-TafE_H458A_ and His_6_-TafE_D461A_, two mutants in which the highly conserved residues His458 and Asp461 in the predicted Ntox15 domain were each replaced with alanine (Fig. 3A and Fig. S4A), and examined their nuclease activities with DNA from different organisms. The inclusion of His_6_-TafE in reaction mixtures containing DNA from E. coli, A. baumannii, or S. cerevisiae led to complete destruction of the large DNA molecules after 60 min of incubation (Fig. 3B). Similar degradation occurred in reaction mixtures containing single-stranded DNA isolated from salmon sperm (Fig. 3B). Both His_6_-TafE_H458A_ and His_6_-TafE_D461A_ had completely lost the ability to degrade DNA (Fig. 3B), indicating that the H_458_XXE_461_ motif is essential for its nuclease activity. As DNases often require specific metal ions for their activity, we first treated His_6_-TafE with EDTA, which abolished its ability to degrade DNA, likely by chelating the metal ion critical for its enzymatic activity. The addition of exogenous Mg^2+^ but not of Ca^2+^ restored its ability to degrade DNA (Fig. 3C). Thus, TafE is a Mg^2+^-dependent DNase. Furthermore, deletion of *tafE* did not detectably impact the ability of A. baumannii to outcompete E. coli, E. cloacae, or S. aureus (Fig. S4B and C), suggesting that the bacterium codes for other effectors to target bacteria.

10.1128/mbio.03420-22.4 FIG S4 Bacterial killing by the Δ*tafE* mutant. (A) Purification of recombinant TafE and its derivates from E. coli. TafE and its derivates with an N-terminal His_6_-sumo tag were introduced into E. coli strain BL21, and the proteins were produced by induction with 0.2 mM IPTG. Soluble fractions obtained by centrifugation were subjected to affinity purification with Ni^2+^ beads, and samples for each step were resolved by SDS-PAGE and detected by Coomassie brilliant blue staining. (B, C) The *tafE* gene is dispensable for A. baumannii to kill bacteria. Each of the indicated strains was mixed with the indicated bacteria at a 1:1 ratio for 4 h. (B) The survival of the prey bacteria was assessed by plating dilutions on selective medium. (C) The results are from three independent experiments, each done in triplicate. Bars and error bars represent mean values for prey survival (CFU) ± standard deviations. Download FIG S4, TIF file, 1.6 MB. Copyright © 2023 Luo et al. 2023 Luo et al. https://creativecommons.org/licenses/by/4.0/ This content is distributed under the terms of the Creative Commons Attribution 4.0 International license.

Unlike bacteria, genomic DNA of fungi is packed in the nucleus, which is surrounded by bilayer membranes with pores that only allow efficient passage of proteins less than about 40 kDa (42). TafE has a predicted molecular weight of approximately 60 kDa and, thus, cannot efficiently enter the nucleus by diffusion. To determine whether TafE can be targeted to the nuclei of yeast cells, we introduced a plasmid expressing green fluorescent protein (GFP)-TafE_H458A_ into yeast strain W303 (43), stained fixed cells with DAPI (4′,6-diamidino-2-phenylindole), and assessed the distribution of the GFP signals under a confocal microscope. In cells expressing GFP-TafE_H458A_, green fluorescence signals perfectly colocalized with those of DAPI, with few green signals being detected in the cytoplasm, whereas GFP-eEF1A, the protein translation elongation factor, was mainly cytosolic, as previously described (Fig. 3D) (44). Intriguingly, GFP-TafE was almost completely localized in the cytoplasm in HeLa cells (Fig. 3D), suggesting that TafE can only be recognized by the nuclear import machine of yeast cells or that the protein can exploit a nuclear translocation mechanism specific to yeast. The cytosolic localization of GFP-TafE is consistent with the observation that this fusion protein was not detectably toxic to HeLa cells, likely due to its inability to reach the substrate in the nucleus (Fig. 3E). These observations suggest that TafE has acquired nuclear localization signals that facilitate its targeting to the nucleus of yeast but not mammalian cells.

### The DNase activity of TafE is essential for A. baumannii to attack fungi.

**FIG 4:**
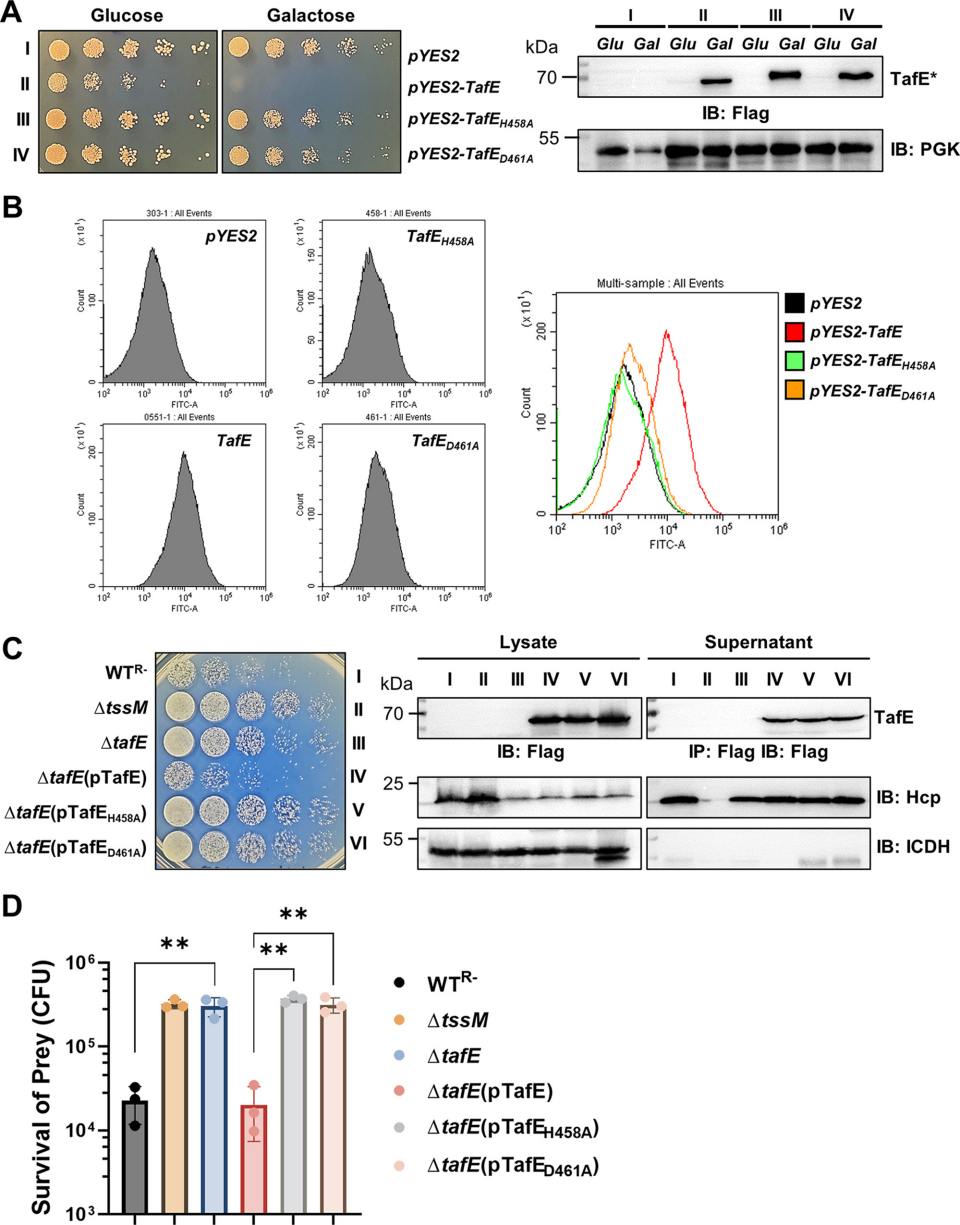
The DNase activity of TafE is essential for the yeast-killing ability of A. baumannii. (A) The HXXD motif is required for yeast toxicity of TafE. Cells of yeast strains harboring plasmids coding for the Flag-tagged TafE or mutants with disrupted HXXD motifs driven by the P_gal_ promoter were spotted onto medium containing glucose or galactose. Left, the growth was assessed by acquiring images after incubation at 30°C for 4 days. Right, the expression of the proteins in each strain was detected in lysates of cells grown in the indicated medium by immunoblotting with Flag-specific antibody. TafE* represents TafE or any of its mutants. (B) Yeast cell death caused by the expression of TafE or its mutants evaluated by fluorescence-activated cell sorting (FACS). After induction with galactose for 12 h, TUNEL-stained cells were analyzed by flow cytometry. (C, D) The integrity of the HXXD motif is important for A. baumannii to kill yeast. The ability of TafE or its HXXD mutants to restore the yeast killing ability of the Δ*tafE* mutant was determined as described in the legend to Fig. 1D. (C) Left, note that mutations in the HXXD motif abolished the ability of TafE to complement the Δ*tafE* mutant. Right, the functionality of the T6SS in indicated strains was determined by their ability to secrete Hcp, and the expression of TafE and its derivates was detected in lysates and culture supernatants by immunoblotting with Flag-specific antibody. (D) The results are from three independent experiments each done in triplicate; bars and error bars represent mean values for prey survival (CFU) ± standard deviations. IB, immunoblotting; IP, immunoprecipitation. **, *P < *0.01.

We further analyzed the role of the DNase activity in the interactions between A. baumannii and fungi. Mutations in His_458_ or Asp_461_ did not affect the stability of TafE in yeast, but both abolished its yeast toxicity (Fig. 4A). Furthermore, because the DNase activity of TafE will cause DNA fragmentation, we examined the presence of DNA termini in yeast cells expressing different alleles of *tafE* using the terminal deoxynucleotidyl transferase-mediated dUTP-biotin nick-end labeling (TUNEL) assay (45). In cells expressing TafE, a population of cells were TUNEL positive. In contrast, DNA labeling did not detectably occur in cells expressing the TafE_H458A_ or TafE_D461A_ mutant (Fig. 4B). These results further validate the DNase activity of TafE.

We next assessed the importance of the DNase activity of TafE in the fungus-killing activity of A. baumannii. Plasmids that direct the expression of TafE, TafE_H458A_, or TafE_D461A_ were introduced into the Δ*tafE* strain, and the ability of the resulting strains to kill yeast was examined. Whereas the expression of TafE fully restored the ability of the Δ*tafE* mutant to kill yeast, neither of the two mutants had such activity (Fig. 4C and D). Thus, the DNase activity of TafE is required for A. baumannii to kill fungi.

### A small fraction of clinical isolates of A. baumannii harbor *tafE*.

The A. baumannii strain 17978 used in our study was isolated in 1951 from a meningitis patient in the United States (27). T6SS effectors are known to be highly diverse in a given bacterial species, which reflects the evolution of the microorganism in its natural habitats (46). We thus set out to determine the distribution of *tafE* in clinical isolates of A. baumannii collected by our hospital. We examined a total of 78 isolates for the *tafE* gene by PCR, using primers corresponding to its 5′ and 3′ ends. Only 3 isolates (no. 11, no. 12, and no. 32) appeared to harbor a *tafE* gene that had sufficient sequence similarity to be detectable by this method (Fig. S5A). Intriguingly, among the three strains, no. 12 and no. 32 expressed and secreted Hcp similarly to strain WT^R−^, a derivative of 17978 lacking pAB3 (Table S1). In contrast, Hcp expression was not detectable in strain no. 11 (Fig. S5B). These results suggest that the T6SS is constitutively expressed in isolates no. 12 and no. 32 but is repressed in isolate no. 11.

10.1128/mbio.03420-22.5 FIG S5 Distribution of *tafE* in clinical isolates of A. baumannii. (A) Detection of *tafE* in clinical isolates of A. baumannii. Genomic DNA of a total of 78 clinical isolates was used as the template to detect *tafE* by PCR. The PCR products were separated by 1% agarose gel electrophoresis and stained using ethidium bromide. Note that the *tafE* gene was detected in three strains (no. 11, no. 12, and no. 32). (B) Hcp secretion profile of the clinical isolates. Culture supernatants of the three strains harboring TafE were probed for Hcp by immunoblotting, and the expression of the gene was evaluated by detecting the protein in total cell lysates of these strains. (C, D) Yeast killing by the three clinical isolates harboring *tafE*. S. cerevisiae strain W303 was used as the prey for each of these three A. baumannii strains in killing assays. Similar results were obtained from at least three independent experiments, and the bar graphs represent mean values for prey survival (CFU) ± standard deviations. *, *P < *0.05; **, *P < *0.01; ns, not significant. Download FIG S5, TIF file, 2.3 MB. Copyright © 2023 Luo et al. 2023 Luo et al. https://creativecommons.org/licenses/by/4.0/ This content is distributed under the terms of the Creative Commons Attribution 4.0 International license.

We next examined the antifungal activity of the three strains using S. cerevisiae as prey. Consistent with the status of Hcp expression and secretion, strains no. 12 and no. 32 efficiently killed yeast cells at rates comparable to the killing by the WT^R−^ strain derived from strain 17978. The antifungal activity of strain no. 11 is akin to that of the Δ*tssM* mutant, suggesting that its T6SS is not active under our experimental conditions (Fig. S5C and D). We also sequenced the *tafE* gene in clinical isolates of A. baumannii, and the results showed that these genes are highly homologous to *ACX60_15365*, each harboring the HXXD motif (Fig. S6). Thus, the presence of TafE endows A. baumannii with the capability to outcompete fungi. Whether any isolate lacking TafE has antifungal activity and, if so, whether such activity is mediated by effectors structurally similar to TafE needs further investigation.

10.1128/mbio.03420-22.6 FIG S6 Sequence alignment of the TafE orthologs in clinical isolates of A. baumannii. The alignment was generated using Jalview (63). Strictly conserved residues are shown in a dark blue background, and residues with weak homology are marked with purple. The HXXD motifs are marked with white letters in a red background. Download FIG S6, TIF file, 1.9 MB. Copyright © 2023 Luo et al. 2023 Luo et al. https://creativecommons.org/licenses/by/4.0/ This content is distributed under the terms of the Creative Commons Attribution 4.0 International license.

### The immunity protein TaeI prevents self-destruction of A. baumannii by TafE.

**FIG 5:**
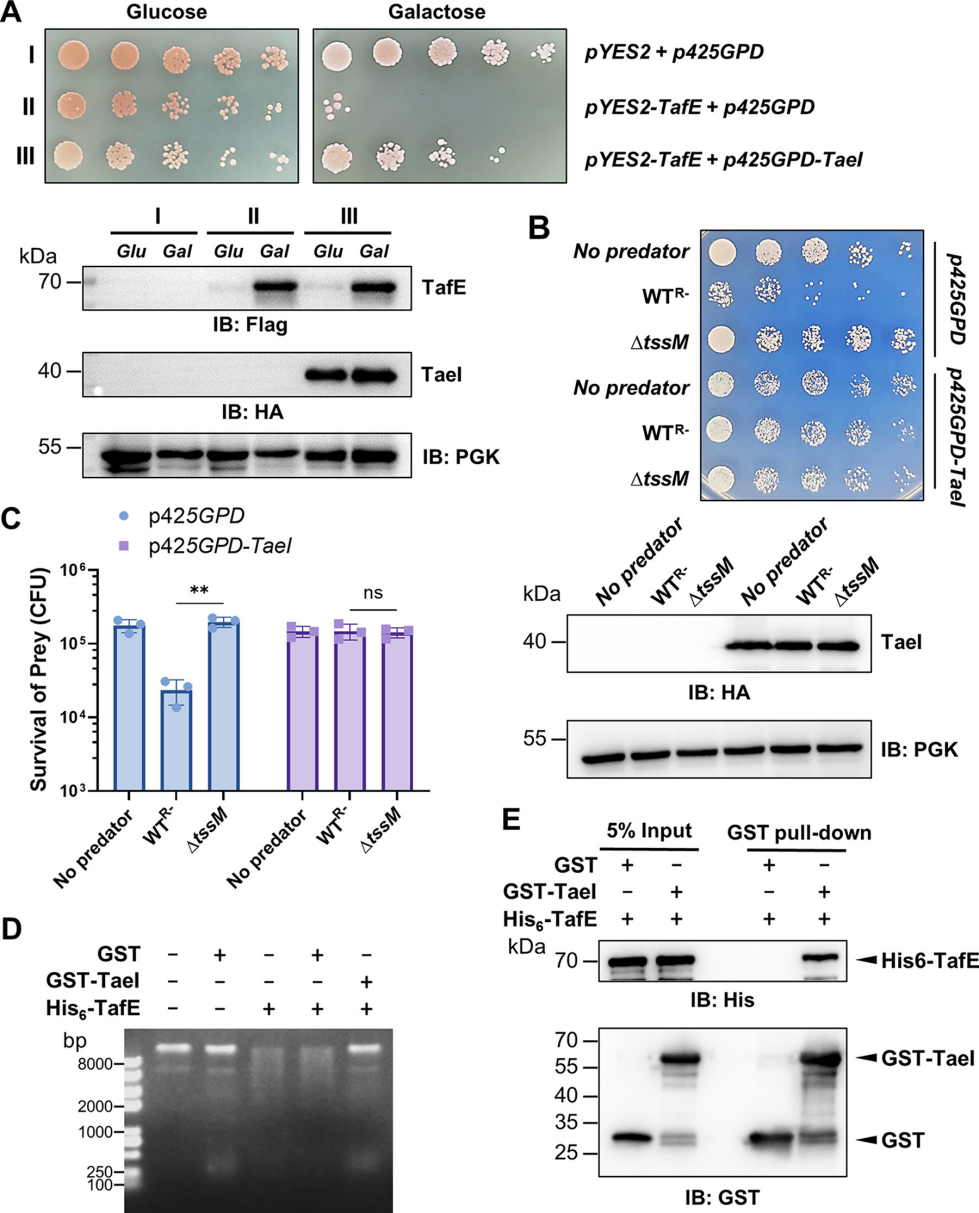
TaeI is the immunity protein that prevents TafE from killing A. baumannii by direct interactions. (A) Coexpression of TaeI blocks the yeast toxicity of TafE. Top, a plasmid constitutively expressing HA-TaeI by the GPD promoter was introduced into the yeast strain harboring Flag-TafE driven by P_gal_. Serially diluted cells of the yeast strains were spotted onto medium containing glucose or galactose, and the growth was assessed after 3 days of incubation. Bottom, the expression of Flag-TafE and HA-TaeI was probed by immunoblotting using antibodies specific for Flag and hemagglutinin (HA), respectively. (B, C) Yeast cells expressing TaeI are resistant to killing by A. baumannii. Yeast strains harboring the HA-TaeI-expressing plasmid or empty vector were used as prey for the indicated A. baumannii strains. (B) Top, survival of yeast after mixing with A. baumannii was determined by spotting serially diluted cells onto selective medium. Images were acquired after 4 days of incubation at 30°C. Bottom, the expression of TaeI in yeast was probed by immunoblotting with HA-specific antibody, and PGK was probed as the loading control. (C) The rates of survival were determined by enumerating the CFU of yeast. The results shown are the mean values for prey survival (CFU) ± standard deviations. Similar results were obtained in three independent experiments. **, *P < *0.01; ns, not significant. (D) TaeI inhibits the DNase activity of TafE. GST or GST-TaeI was mixed with TafE, and DNA was added to the reaction mixtures. The integrity of the DNA was evaluated by agarose gel electrophoresis. Note that the DNA remained intact in reaction mixtures receiving both His6-TafE and GST-TaeI. (E) TaeI directly interacts with TafE. GST or GST-TaeI was mixed with His_6_-TafE, and the protein complex was captured by GST beads. Note that His_6_-TafE was pulled down by the beads in the presence of GST-TaeI.

The indiscriminate DNase activity of TafE against DNA from different organisms, including A. baumannii itself, immediately suggests the presence of an immunity protein that functions to prevent self-destruction by TafE. Such immunity proteins, which often are encoded by genes next to those of the effectors, have been described for many lethal T6SS effectors (47, 48). In strain 17978, the open reading frame *ACX60_15360* immediately downstream from *tafE* that is predicted to code for a protein of 286 residues is a good candidate for the immunity protein against TafE (Fig. S1). We first determined whether TaeI could suppress the yeast toxicity of TafE. Coexpression of TaeI with TafE abolished the toxicity of the effector and allowed yeast to grow (Fig. 5A). The expression of mCherry-TaeI in yeast revealed that the protein occupies the entire cell with a clear concentration in the nucleus (Fig. S7A), suggesting that TaeI may engage TafE before and after it has reached the nuclei of target fungal cells. Furthermore, yeast cells harboring a plasmid expressing TaeI became resistant to the WT^R−^ strain of A. baumannii, which killed a control strain carrying the empty vector (Fig. 5B and C). These results indicate that TaeI antagonizes the toxicity of TafE in A. baumannii and protects the bacterium from the lethal effect of the indiscriminate DNase activity.

10.1128/mbio.03420-22.7 FIG S7 Cellular localization of TaeI in yeast, its purification, and inhibition of TafE’s toxicity to E. coli. (A) TaeI is distributed throughout the yeast cells. Cells of the S. cerevisiae strain expressing mCherry-TaeI were fixed and stained with DAPI. Cellular localization of the fusion protein was assessed by confocal microscopy. Note that TafE occupies the entire cell with a clear concentration in the nucleus. (B) Purification of recombinant TaeI from E. coli. The recombinant TaeI with an N-terminal GST tag was induced to express in E. coli with 0.2 mM IPTG. Soluble fractions were used for protein purification; samples at each step of the purification procedure were analyzed by SDS-PAGE, and proteins were detected by Coomassie brilliant blue staining. (C, D) Coexpression of TaeI suppresses the toxicity of TafE to bacteria. GST-TaeI was introduced into strain BL21(DE3) expressing His_6_-sumo-TafE. Bacterial growth was assessed by spotting dilutions of cell suspensions onto LB plates with and without IPTG. (D) The expression of TafE and TaeI after IPTG induction was determined by immunoblotting with anti-His_6_ and anti-GST antibodies. Download FIG S7, TIF file, 0.02 MB. Copyright © 2023 Luo et al. 2023 Luo et al. https://creativecommons.org/licenses/by/4.0/ This content is distributed under the terms of the Creative Commons Attribution 4.0 International license.

We further explored the mechanism of action of TaeI by examining its effect on the DNase activity of TafE. Glutathione *S*-transferase (GST)-tagged TaeI was purified from E. coli (Fig. S7B), and the inclusion of TaeI in reaction mixtures at a 1:1 molar ratio to TafE effectively abolished its DNA degradation activity (Fig. 5D). Most immunity proteins block the activity of the cognate effectors by direct protein-protein interactions (47), and thus, we examined binding between TafE and TaeI. In solutions containing GST-TaeI and His_6_-TafE, the latter could be captured by GST beads together with GST-TaeI, which did not occur in reaction mixtures containing GST and His_6_-TafE (Fig. 5E), indicating that these two proteins form a stable complex. In addition, coexpressed TaeI also suppressed the bacterial toxicity of TafE in E. coli (Fig. S7C and D). Together, these results indicate that TaeI is the immunity protein of TafE, which protects A. baumannii from the toxicity of the effector by direct binding.

## DISCUSSION

The ability of A. baumannii to thrive in nutrient-scarce niches and to outcompete other microbes in polymicrobial communities depends on its mechanisms for effective nutrient assimilation and killing of competitors. The discovery of TafE as a weapon against fungi and Gram-negative and Gram-positive bacteria by A. baumannii further underpins its success in harsh environments. More importantly, effectors with potent toxicity to the bacterium itself, such as TafE, may be exploited to develop novel and specific antibiotics by eliminating the immunity proteins using technologies such as BacPROTACs (49).

Tse4 of A. baumannii has both lytic transglycosylase and endopeptidase activities that attack the cell wall of both Gram-positive and Gram-negative bacteria (29). In addition, Tae and Tle are likely antibacterial effectors, given their strong toxicity toward E. coli (Fig. S1). Thus, A. baumannii utilizes multiple effectors to attack bacteria, which is a common phenomenon among different bacteria (5). The cell wall is considered the major barrier for the penetration of the T6SS spear. The cell wall of yeast (>110 nm) is considerably thicker than that of Gram-positive bacteria (<80 nm) (15, 50), and thus, the ability to kill fungi suggests that A. baumannii’s T6SS is more penetrative than those of bacteria lacking this ability. Given the nonspecific targeting of DNA by TafE, it may also contribute to the killing of bacteria. At the same time, this effector appears to have evolved to target fungal cells because of its targeting to the nuclei of yeast but not mammalian cells, probably by nucleus localization signals specific for the former (Fig. 3). Whether TafE provides advantages to A. baumannii for its infections in humans and animals remains to be investigated.

Although their biochemical activities remain elusive, Tfe1 and Tfe2 from S. marcescens cause fungal cell death by inducing the formation of abnormally large vacuoles and by disruption of fungal metabolism, respectively (16). In addition, M. xanthus utilizes the proline-alanine-alanine-arginine (PAAR) protein to kill plant-pathogenic fungi (18). Despite the apparent importance of killing yeast in its competition for space and nutrients, it is surprising that only 3 of the 78 A. baumannii clinical isolates detectably carry the *tafE* gene (Fig. S5). Other isolates may employ effectors with activities distinct from that of TafE to kill fungi. It is important to note that not all bacteria that code for effectors with nuclease activity can kill yeast or Gram-positive bacteria, suggesting that the penetrative power of the T6SS differs among bacterial species. Alternatively, for bacteria that can kill fungi, other yet-unknown factors may facilitate the penetration of their T6SS across the thick cell wall.

The antimicrobial function of T6SS is critically important in overcoming the resistance to colonization by pathogens that need to establish a niche in polymicrobial environments like the gut (5). A. baumannii clearly will encounter other microbes when it attempts to establish a niche, whether on the surface of medical devices, the skin, or a wound. It is not clear how the T6SS and its effectors impact A. baumannii virulence under natural conditions when other microbes are present. Better infection models that can more closely recapitulate the infection process in the presence of other microbes are needed to assess the role of the T6SS and its effectors in interactions between A. baumannii and its hosts. Such information will drive further research to explore strategies to inactivate the T6SS to prevent and treat infections caused by this increasingly challenging pathogen.

## MATERIALS AND METHODS

### Bacterial strains and growth conditions.

The bacterial and yeast strains used in the study are listed in Table S1. With the exception of the 78 clinical isolates used to probe the distribution of the *tafE* gene, all A. baumannii strains were derived from strain 17978 (27). Strains of E. cloacae, S. aureus, C. albicans, and C. glabrata were isolates recovered from patients in the First Hospital of Jilin University, Changchun City, Jilin Province, China. Each was verified by next-generation sequencing (NGS). Unless otherwise noted, bacteria were grown in Luria-Bertani medium. For E. coli, antibiotics were used at the following concentrations: ampicillin, 100 μg/mL, and kanamycin, 30 μg/mL. For A. baumannii, the concentrations were gentamicin, 10 μg/mL, and streptomycin, 100 μg/mL.

For S. cerevisiae, strain W303 (43) was used. The yeast strains and clinical fungal isolates were grown in YPD (1% yeast extract, 2% peptone, 2% glucose) or Sabouraud dextrose (SD) minimal medium containing nitrogen base, glucose, and amino acid drop-out mix for selection of transformed plasmids as described previously (51). To induce gene expression from the galactose promoter, 2% galactose was included in synthetic medium.

### Isolation of a derivative of A. baumannii strain 17978 lacking plasmid pAB3.

A. baumannii strain 17978 harbors pAB3, a large conjugative plasmid that codes for two *tetR*-like genes that repress the expression of the chromosomal T6SS (28, 52). This plasmid is known to be unstable and can be cured as the bacterium propagates (28). We randomly tested 200 colonies of strain 17978 by PCR using primer pairs pSL1041/pSL1042 and pSL1043/pSL1044 to amplify two fragments on pAB3 (accession no. CP012005), from which one strain negative for these two genes was obtained. After verification with the third primer pair, pSL1045/pSL1046, the strain that had lost pAB3, designated WT^R−^, was chosen for subsequent experiments.

### Construction of plasmids and bacterial mutants.

Bioinformatic analysis of strain A. baumannii 17978 revealed the presence of four VgrG clusters, each associated with a putative effector, Tle, TafE, Tse, and Tae, respectively (Fig. S1). The genes encoding Tle, TafE, Tse, and Tae were each deleted from the genome of strain 17978 as described previously (53). Briefly, the deletion plasmid was constructed by amplifying 0.8-kb DNA fragments upstream and downstream from each gene and ligating them into SacI-/SalI-digested pSR47s, an R6K-based suicide plasmid (54). This manipulation replaced the gene to be deleted with an open reading frame that encoded a 15-residue polypeptide composed of the first 15 and last 15 amino acids of the original protein. The resulting gene deletion plasmid was introduced into a streptomycin-resistant derivative of strain 17978 (39) by triparental mating with the help of E. coli strain MT607 (pRK600) (55). Transconjugants were streaked onto LB agar containing 5% sucrose to select cells in which the second event of recombination had occurred. Mutants with the appropriate deletion were identified by primer pairs corresponding to the gene of interest by PCR. The integrity of all plasmids was verified by sequencing analysis. The sequences of all primers used in this study are in Table S1.

### Protein expression and purification.

The full-length gene for TafE was amplified from A. baumannii genomic DNA using the appropriate primers (Table S1), and the PCR product was digested with BamHI/SalI and inserted into pETSumo (Thermo Fisher) to produce sumo-His_6_-tagged fusion proteins. The gene coding for TaeI, an immunity protein that antagonizes the activity of TafE, was cloned into pGEX-6P-1 to express GST-TaeI. When needed, substitution mutations were introduced using the QuikChange kit (Agilent) and fusion PCR. E. coli strain BL21(DE3) was used as the host for expression and purification of recombinant proteins.

Amounts of 20 mL of overnight cultures were transferred to 500 mL LB broth supplemented with 100 μg/mL of ampicillin or 30 μg/mL of kanamycin. Cultures were grown to an optical density at 600 nm (OD_600_) of 0.6 in a shaker (200 rpm) at 37°C. The expression of the fusion protein was induced at 16°C by adding 0.2 mM IPTG (isopropyl-β-d-thiogalactopyranoside) for 18 h. The harvested cell pellets were resuspended in lysis buffer (50 mM NaH_2_PO_4_, 300 mM NaCl, 10 mM imidazole) and were lysed by using a cell homogenizer (JN-mini; JNBio, Guangzhou, China). The supernatant of lysed cells was mixed with Ni^2+^-nitrilotriacetic acid (NTA) or glutathione beads at 4°C for 1 h. Ni^2+^-NTA resin was extensively washed with washing buffer (50 mM NaH_2_PO_4_, 300 mM NaCl, 20 mM imidazole) following protein binding. Glutathione beads were washed with 20× the column volume of phosphate-buffered saline (PBS). Bound His_6_-sumo-tagged proteins were eluted five times with the same buffer containing 250 mM imidazole. GST-tagged proteins were eluted with 10 mM glutathione. His_6_-Hcp and His_6_-TafE fusion proteins were similarly purified with Ni^2+^-NTA beads. The purity of the proteins was evaluated by SDS-PAGE followed by Coomassie brilliant blue staining, and only proteins purer than 95% were used in subsequent experiments. Proteins were dialyzed in a storage buffer (50 mM Tris·HCl, 150 mM NaCl, 10% glycerol) at 4°C. Protein concentration was determined by the Bradford method.

### Yeast manipulation.

The yeast strains used in this study were derived from strain W303 (43), which was grown at 30°C in YPD medium. For maintenance of plasmids, yeast cells were cultured in an appropriate amino acid dropout synthetic medium supplemented with 2% glucose or galactose as the sole carbon source (56). For assessment of yeast toxicity, each of the putative A. baumannii T6SS substrates or TafE mutants was cloned into pYES2/NTA (Invitrogen), which harbors a galactose-inducible promoter controlling its expression (57). The plasmids were introduced into yeast cells using the lithium acetate method (58). Overnight cultures were serially diluted (5-fold), and 10 μL of each dilution was spotted onto selective plates containing glucose or galactose. The plates were incubated at 30°C for 2 days before image acquisition.

### Bacterial and fungal competition assay.

To determine the bacterial killing activity of A. baumannii, interbacterial competition assays were performed between A. baumannii and E. coli, E. cloacae, or S. aureus as described previously (29). Briefly, the bacterial cells were grown overnight in LB broth at 37°C. Cells washed 2× with sterile PBS were resuspended with fresh LB broth. A. baumannii and the prey bacteria with kanamycin resistance were mixed at a ratio of 1:1 (A. baumannii/E. coli *or* A. baumannii/E. cloacae) or 1:10 (A. baumannii/S. aureus), and 10 μL of the mixture was spotted on LB agar and incubated for 4 h at 37°C. The number of viable cells was determined by plating serially diluted cells on LB agar with kanamycin and culturing for another 18 h before image acquisition and colony counting. The number of input cells in each case was similarly determined using diluted cells that never met the predator strain. To evaluate the antifungal activity of A. baumannii, the yeast strain W303 and clinical isolates C. albicans and C. glabrata were used as the prey organisms. Similar procedures were used for the A. baumannii-and-fungal-prey competition assays, except that the cell mixtures were coincubated at a ratio of 10:1 (predator/prey) at 37°C for 6 h and grown on YPD plates containing 30 μg/mL kanamycin for 3 days before image acquisition and CFU counting.

### Bacterial growth inhibition assay.

To test the potential bacterial toxicity, each of the indicated putative T6SS substrates of A. baumannii was introduced into the pET-sumo plasmid, which was introduced into E. coli strain BL21(DE3). To assess the effects of *taeI* on the bacterial toxicity of TafE, the gene coding for the immunity protein was cloned into pGEX-6p1 and the resulting plasmid was transformed into BL21(DE3) harboring pET-sumo-TafE. Cells were grown in LB medium at 37°C overnight. After being adjusted to the same OD_600_ value, the cultures were serially diluted (10-fold) and 10 μL of each dilution was spotted onto selective plates with or without 0.2 mM IPTG. The uninduced plates were cultured at 37°C for 18 h, and the plates containing IPTG were incubated at 25°C for 2 days due to IPTG induction potentially influencing bacterial growth at 37°C.

### TUNEL staining and flow cytometry analysis.

Yeast strains derived from W303 and harboring a plasmid expressing TafE or its derivatives were grown at 30°C in selective liquid medium with 2% raffinose overnight. After washing twice with PBS, the yeast cells were resuspended in medium containing 2% galactose and continuously cultured at 30°C for another 8 h. A TUNEL staining assay was performed to determine the DNA damage in yeast according to a previously reported procedure (59). Briefly, collected cells were fixed with 3.7% (vol/vol) formaldehyde for 30 min at room temperature and washed with PBS 3 times. After being digested with Zymolyase (MP Biomedicals) at 37°C for 1 h, cells were rinsed and incubated with 0.3% Triton X-100 for 5 min, followed by staining using a one-step TUNEL cell apoptosis detection kit (Beyotime Biotechnology, China). Then, the collected cells were analyzed by flow cytometry (Beckman CytoFLEX) at a 515- to 565-nm excitation wavelength and a 450- to 500-nm emission wavelength.

### Antibodies and immunoblotting.

Purified His_6_-Hcp and His_6_-TafE were used to immunize rabbits by standard procedures (Jiaxuan Biotechnology Co., Ltd., Beijing, China). We used rabbit anti-Hcp (1:2,000), rabbit anti-isocitrate dehydrogenase (ICDH) (1:2,000) (39), mouse anti-Flag (1:3,000) (catalog number F1804; Sigma), and mouse anti-phosphoglycerate kinase (PGK) (1:2,500) (catalog number ab113687; Abcam) antibodies in standard immunoblotting procedures. Briefly, proteins extracted from cells with Laemmli buffer were resolved by SDS-PAGE. Separated proteins were transferred onto polyvinylidene fluoride (PVDF) membranes (Pall Life Sciences), and the membranes were first blocked with 5% nonfat milk (Bio-Rad) and then incubated with the appropriate primary antibodies at 4°C for 14 h. After being washed 3 times, the membranes were incubated with appropriate IRDye- or horseradish peroxidase (HRP)-labeled secondary antibodies, and the signals were detected and analyzed by using an Odyssey CLx system (LI-COR) or ChemiDoc MP system (Bio-Rad).

### Immunoprecipitation.

To test the T6SS-dependent secretion by A. baumannii, TafE was cloned into pJL03 (39) to express Flag-TafE, and the plasmid was introduced into the WT^R−^, Δ*tssM*, and Δ*vgrG2* strains. Cultures of each strain induced by 1% arabinose for 6 h were centrifuged at 10,000 × *g* for 5 min, and the supernatant was further centrifuged at 15,000 × *g* for 30 min at 4°C. Beads coated with Flag-specific antibody (catalog number F2426; Sigma) were washed twice with PBS and then mixed with the prepared cell supernatant. The mixture was incubated on a rotatory shaker at 4°C for 14 h. After 5 washes with PBS, the resin was boiled in Laemmli buffer at 95°C for 10 min to release the bound proteins.

### Transfection and fluorescence imaging.

To determine the subcellular localization in mammalian cells, GFP-TafE was expressed in HeLa cells by transfection using Lipofectamine 3000 (catalog number L3000150; Invitrogen) according to the manufacturer’s protocol. Twenty-four hours after transfection, cells were stained with Hoechst 33342 (1:5,000) (catalog number H3570; Invitrogen) to label the nuclei. Fluorescence images of the samples were acquired using an Olympus IX-83 fluorescence microscope.

To assess its distribution in yeast, the *gfp*-*tafE*_H545A_ fusion protein gene was inserted into p425GPD (60), and the resulting plasmid was introduced into yeast strain W303. Amounts of 333 μL of cells of the strain harboring the plasmid were mixed with 666 μL of ethanol, and the mixture was incubated at room temperature (25°C) for 1 h. The yeast cells were spun down, and the pellet was suspended with PBS. After staining with Hoechst 33342 (1:2,000), samples were observed under a fluorescence microscope for image acquisition.

### CCK8 assay for assessing cell viability.

To assess the toxicity of TafE to mammalian cells, the CCK8 assay was performed in HeLa cells expressing GFP or GFP-TafE, as described previously (61). Briefly, HeLa cells were seeded into 96-well plates at a density of 2,000 cells/well and cultured for 14 h. An amount of 10 μL of CCK8 solution (Beyotime Biotechnology) was added into each well, and the plate was incubated at 37°C for 1 h. The absorbance of cells was measured at 450 nm using the BioTek Synergy H1 microplate reader to determine cell viability.

### DNase activity assay.

Genomic DNA isolated from bacterial or yeast cells using the TIANamp DNA kit (Tiangen Biotech, China) and salmon sperm DNA procured from Solarbio Science & Technology Co., Ltd. (Beijing, China), were used as the substrates in the DNase activity assay. One microgram of DNA was incubated with purified TafE or its derivatives (1 μg) in 50 μL of reaction buffer (20 mM NaCl, 10 mM Tris-HCl, 0.2 mM dithiothreitol) for 1 h at 37°C in the presence or absence of 2 mM MgCl_2_. The integrity of DNA was analyzed by 1% agarose gel electrophoresis, and images were taken using the Tanon mini space series gel image analysis system.

### GST pull down and immunoprecipitation.

GST pulldown assays were performed as described previously (62). Briefly, 50 μg His_6_-TafE was added into PBS containing 50 μg GST or GST-TaeI, and the proteins were gently mixed and incubated at 4°C for 4 h. An amount of 50 μL of GST beads was added to the reaction mixture and incubated for 1 h. The beads were then separated and washed 5 times with PBS containing 500 mM NaCl to remove unbound proteins. Before SDS-PAGE separation and detection, proteins associated with beads were solubilized in 50 μL of Laemmli buffer. Retained proteins were detected by immunoblot coated with the His_6_-specific antibody after SDS-PAGE.

### Bioinformatics analysis.

Toxin_43 domain-containing proteins were identified using the Pfam database, and multiple sequence alignments (MSAs) of the toxin_43 domain-containing proteins (Atu4350, N643_13510, VPUCM_729, and NM96_04490) from different species were performed using Jalview (63). TafE homologs were identified with tBLASTn using the TafE protein (*ACX60_15365*) from strain ATCC 17978 as the query sequence. The TafE homologs obtained from *Alphaproteobacteria* (genera Methylobacterium, Vannielia, Tritonibacter, Paracoccus, Jannaschia, Loktanella, and Rhodovulum), *Betaproteobacteria* (genera Paraburkholderia and Burkholderia), and *Gammaproteobacteria* (genera Vibrio, Pseudoalteromonas, Acinetobacter, and Pseudomonas) were aligned by using ClustalX 2.1, and phylogenies were constructed using MEGA 7.0 (64) with 1,000 replicates using the neighbor-joining (NJ) method. The Interactive Tree of Life (iTOL) was used for visualization of the phylogenetic tree (65).

### Statistical analyses.

Quantitative data were processed and analyzed using GraphPad Prism 9 software (GraphPad Prism, San Diego, CA, USA). Student’s *t* test was used to compare the mean levels between two groups, each with at least three independent samples.
