# Supplementary material for: Acinetobacter baumannii Kills Fungi via a Type VI DNase Effector
Source: mBio. 2023 Jan 10;14(1):e03420-22. doi: 10.1128/mbio.03420-22 (PMC9973263; doi:10.1128/mbio.03420-22)
Supplement: TABLE S1 [file mbio.03420-22-s0008.docx]

**Table S1 Bacterial strains, plasmids and primers used in this study**

| **Bacterial Strains** | **Source** | **Identifier** |
| --- | --- | --- |
| *A. baumannii* 17978 | (1) | N/A |
| *A. baumannii* WT^R-^ | This study | N/A |
| ∆*tssM* | This study | N/A |
| ∆*tafE* | This study | N/A |
| ∆*tafE*(pJL03) | This study | N/A |
| ∆*tafE*(pTafE) | This study | N/A |
| ∆*tafE*(pTafE_H458A_) | This study | N/A |
| ∆*tafE*(pTafE_D461A_) | This study | N/A |
| ∆*ACX60_17660* | This study | N/A |
| ∆*ACX60_11695* | This study | N/A |
| ∆*ACX60_00605* | This study | N/A |
| WT^R-^(pTafE) | This study | N/A |
| ∆*tssM*(pTafE) | This study | N/A |
| ∆*vgrG2*(pTafE) | This study | N/A |
| *A. baumannii* Clinical isolate #11 | This study | N/A |
| *A. baumannii* Clinical isolate #12 | This study | N/A |
| *A. baumannii* Clinical isolate #32 | This study | N/A |
| *E. cloacae* (Clinical isolate) | This study | N/A |
| *S.* *aureus* (Clinical isolate) | This study | N/A |
| *C. albicans* (Clinical isolate) | This study | N/A |
| *C. glabrata* (Clinical isolate) | This study | N/A |
| *S.* cerevisiae(W303) | (2) | N/A |
| *E. coli* BL21(DE3) | TransGen | CAT# CD601 |
| *E. coli* XL1-Blue | TransGen | CAT# CD401 |

| **Plasmids** | **Source** | **Identifier** |
| --- | --- | --- |
| pJL03 | (3) | N/A |
| pJL03::*tafE* | This study | N/A |
| pJL03::*tafE*_H458A_ | This study | N/A |
| pJL03::*tafE*_D461A_ | This study | N/A |
| pGEX6p-1 | Cytiva | CAT#28-9546-48 |
| pGEX6p-1::*taeI* | This study | N/A |
| pET SUMO | ThermoFisher | CAT#K30001 |
| pET SUMO::*tafE* | This study | N/A |
| pET SUMO::*tafE*_H458A_ | This study | N/A |
| pET SUMO::*tafE*_D461A_ | This study | N/A |
| pET SUMO::*ACX60_17660* | This study | N/A |
| pET SUMO::*ACX60_11695* | This study | N/A |
| pET SUMO::*ACX60_00605* | This study | N/A |
| pYES2CT | Invitrogen | CAT#V825120 |
| pYES2CT::*tafE* | This study | N/A |
| pYES2CT::*tafE*_H458A_ | This study | N/A |
| pYES2CT::*tafE*_D461A_ | This study | N/A |
| pYES2CT::*ACX60_17660* | This study | N/A |
| pYES2CT::*ACX60_11695* | This study | N/A |
| pYES2CT::*ACX60_00605* | This study | N/A |
| p425GPD | (4) | N/A |
| p425GPD::*taeI* | This study | N/A |
| p425GPD::*GFP-TafE_H458A_* | This study | N/A |
| pEGFP-C1 | Clontech | CAT#6084-1 |
| GFP::*tafE* | This study | N/A |
| GFP::*hsa-ran* | This study | N/A |

| **Primers** | **Sequence (Restriction enzyme sites are underlined)** | **Note** |
| --- | --- | --- |
| pSL1001 | cgcggatccatgccagatcctcaaaat | *tafE* up BamHI |
| pSL1002 | acgcgtcgacttacttacaacgcttcaatt | *tafE* down SalI |
| pSL1003 | cgcggatccatgccagatcctcaaaat | *ACX60_15370* up *Bam*HI |
| pSL1004 | acgcgtcgacttacttacaacgcttcaatt | *ACX60_15370* down *Sal*I |
| pSL1005 | cgcggatccatgaaccaagaagatattgattacttttat | *taeI* up *Bam*HI |
| pSL1006 | acgcgtcgactcagttctcaccatccca | *taeI* down *Sal*I |
| pSL1007 | cgcggatccatgaatccttatactatttttgaatat | *ACX60_11695* up *Bam*HI |
| pSL1008 | acgcgtcgacttacacattatatgtttcatatttatct | *ACX60_11695* down *Sal*I |
| pSL1009 | cgcggatccatggataatgtaatagatgcc | *ACX60_17660* up *Bam*HI |
| pSL1010 | acgcgtcgactcattttattctactcaactctt | *ACX60_17660* down *Sal*I |
| pSL1011 | cgcggatccatgagtaatatacatatagtcaaaaaag | *ACX60_00605* up *Bam*HI |
| pSL1012 | acgcgtcgacttacctatttactttaacaccatt | *ACX60_00605* down *Sal*I |
| pSL1013 | ccacctaattgcatatctggattagctaagacatctaatccttctaacgt | *tafE*_H458A_-1 |
| pSL1014 | acgttagaaggattagatgtcttagctaatccagatatgcaattaggtgg | *tafE*_H458A_-2 |
| pSL1015 | catcaaaaccacctaattgcatagctggattatgtaagacatctaat | *tafE*_D461A_-1 |
| pSL1016 | attagatgtcttacataatccagctatgcaattaggtggttttgatg | *tafE*_D461A_-2 |
| pSL1017 | ctggagctcctttgaactcagaactgaccag | *tafE*-KO-A-F *Sac*I |
| pSL1018 | ctttccccatttctgcgatctttgaccag | *tafE*-KO-A-R |
| pSL1019 | gatcgcagaaatggggaaagatgcgatg | *tafE*-KO-B-F |
| pSL1020 | acgcgtcgaccttaacgctccagcatttca | *tafE*-KO-B-R *Sal*I |
| pSL1021 | ctggagctcggcgctaaaatgtttccac | *ACX60_15370*-KO-A-F *Sac*I |
| pSL1022 | gttgagtaaaaaaaacctgactattcaggga | *ACX60_15370*-KO-A-R |
| pSL1023 | tcaggttttttttactcaacttggaagcagtac | *ACX60_15370*-KO-B-F |
| pSL1024 | acgcgtcgacgtggtggaggtgtttctt | *ACX60_15370*-KO-B-R *Sal*I |
| pSL1025 | ctggagctcagccatccaaaagacaag | *ACX60_17660*-KO-A-F *Sac*I |
| pSL1026 | cctgcacactcgcttgtttttttatatctttattgg | *ACX60_17660*-KO-A-R |
| pSL1027 | aaaacaagcgagtgtgcaggatattttgt | *ACX60_17660*-KO-B-F |
| pSL1028 | acgcgtcgactctttcaggctcaattgtatatt | *ACX60_17660*-KO-B-R *Sal*I |
| pSL1029 | ctggagctcaacttgggtaacttaagcc | *ACX60_11695*-KO-A-F *Sac*I |
| pSL1030 | cttgatcaaaattataaaagactattgtatattcaaaaatagt | *ACX60_11695*-KO-A-R |
| pSL1031 | cttttataattttgatcaagacgatacagat | *ACX60_11695*-KO-B-F |
| pSL1032 | acgcgtcgacaagatgattagacagaatttagc | *ACX60_11695*-KO-B-R *Sal*I |
| pSL1033 | ctggagctccaagtattaaagaccgaattcat | *ACX60_00605*-KO-A-F *Sac*I |
| pSL1034 | acttaatcatcttatcccccttattatttatcaaa | *ACX60_00605*-KO-A-R |
| pSL1035 | gggggataagatgattaagtataaattatgtctggca | *ACX60_00605*-KO-B-F |
| pSL1036 | acgcgtcgaccaattttcgaaataatatttttatcgc | *ACX60_00605*-KO-B-R *Sal*I |
| pSL1037 | tgcatcaccaattcaaattttctaa | pAB3-1-F |
| pSL1038 | atgaaaatccgccttatttgcgctta | pAB3-1-R |
| pSL1039 | atggagtttgaaatcatgactaaa | pAB3-2-F |
| pSL1040 | ctttctttaaagcttgttgggcc | pAB3-2-R |
| pSL1041 | tgctgtctctttttatccgtctt | pAB3-3-F |
| pSL1042 | taacagcgtaccgttatatcc | pAB3-3-R |

N/A, not applicable.

1. Piechaud M, Second L. 1951. [Studies of 26 strains of Moraxella Iwoffi]. Ann Inst Pasteur (Paris) 80:97-9.

2. Fan HY, Cheng KK, Klein HL. 1996. Mutations in the RNA polymerase II transcription machinery suppress the hyperrecombination mutant hpr1 delta of Saccharomyces cerevisiae. Genetics 142:749-59.

3. Jie J, Chu X, Li D, Luo Z. 2021. A set of shuttle plasmids for gene expression in Acinetobacter baumannii. PLoS One 16:e0246918.

4. Mumberg D, Muller R, Funk M. 1995. Yeast vectors for the controlled expression of heterologous proteins in different genetic backgrounds. Gene 156:119-22.
