# Supplementary figures and images for: Acinetobacter baumannii Kills Fungi via a Type VI DNase Effector
Source: mBio. 2023 Jan 10;14(1):e03420-22. doi: 10.1128/mbio.03420-22 (PMC9973263; doi:10.1128/mbio.03420-22)

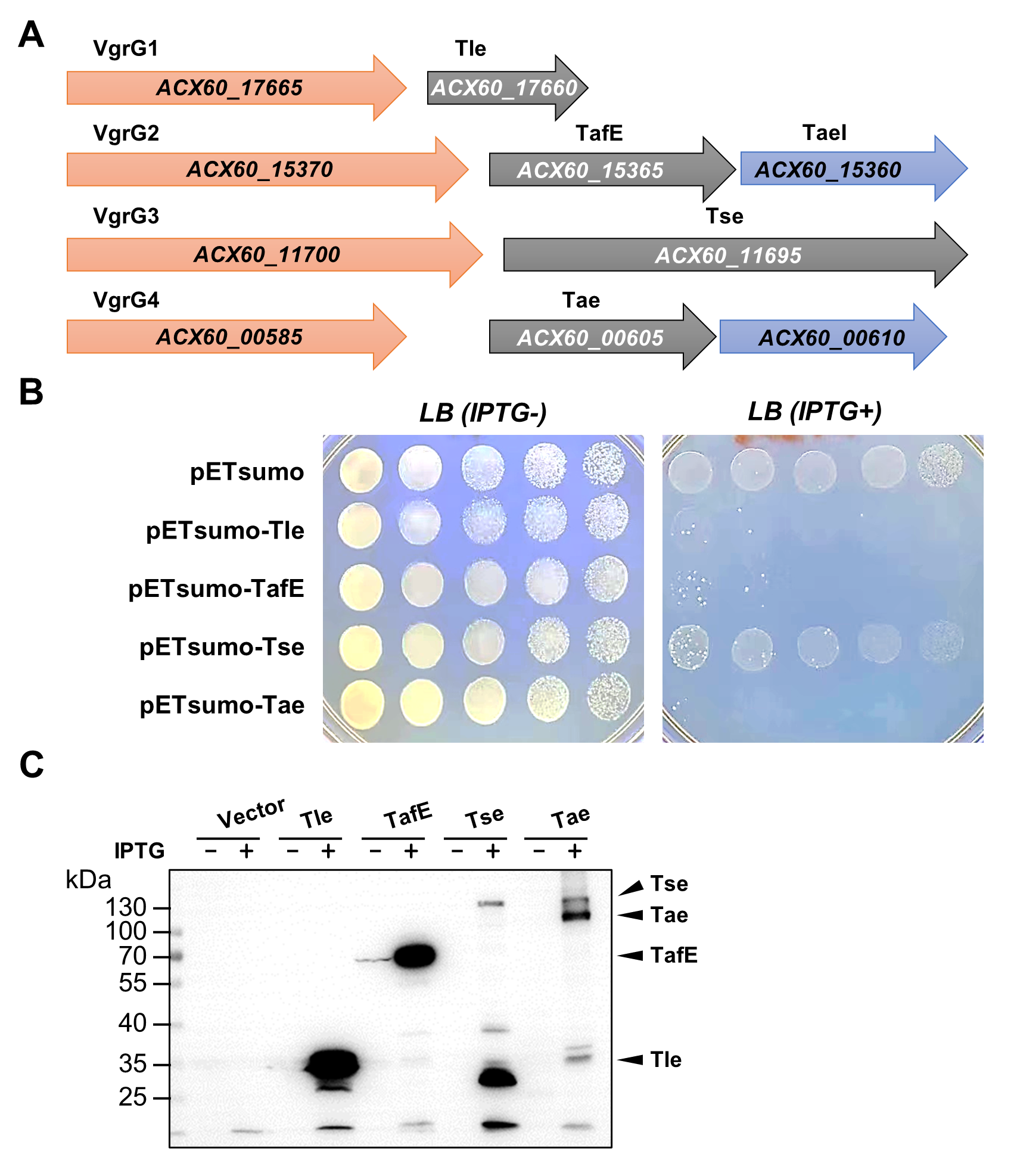

Supplement: FIG S1 [file mbio.03420-22-s0001.tif]

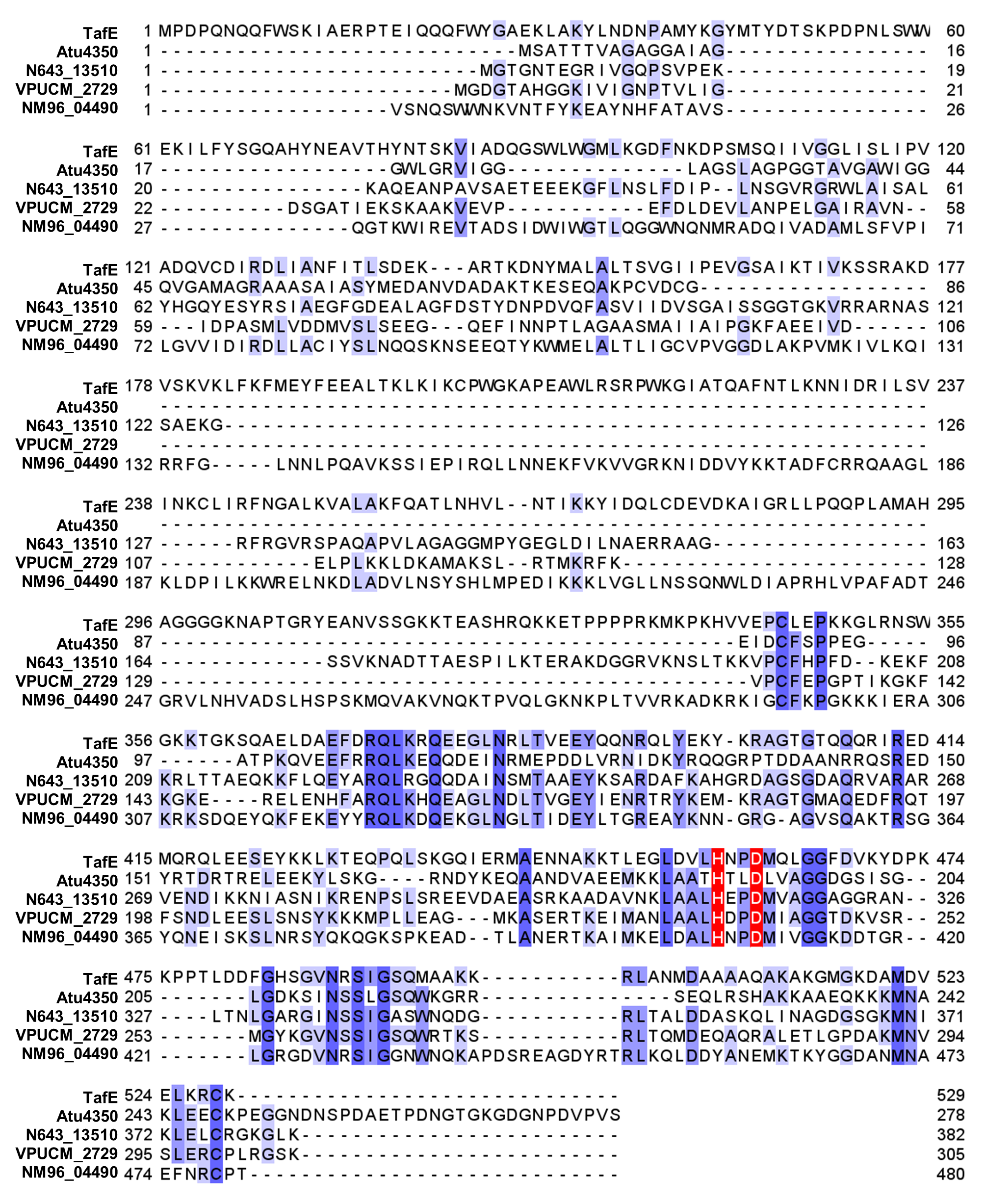

Supplement: FIG S2 [file mbio.03420-22-s0002.tif]

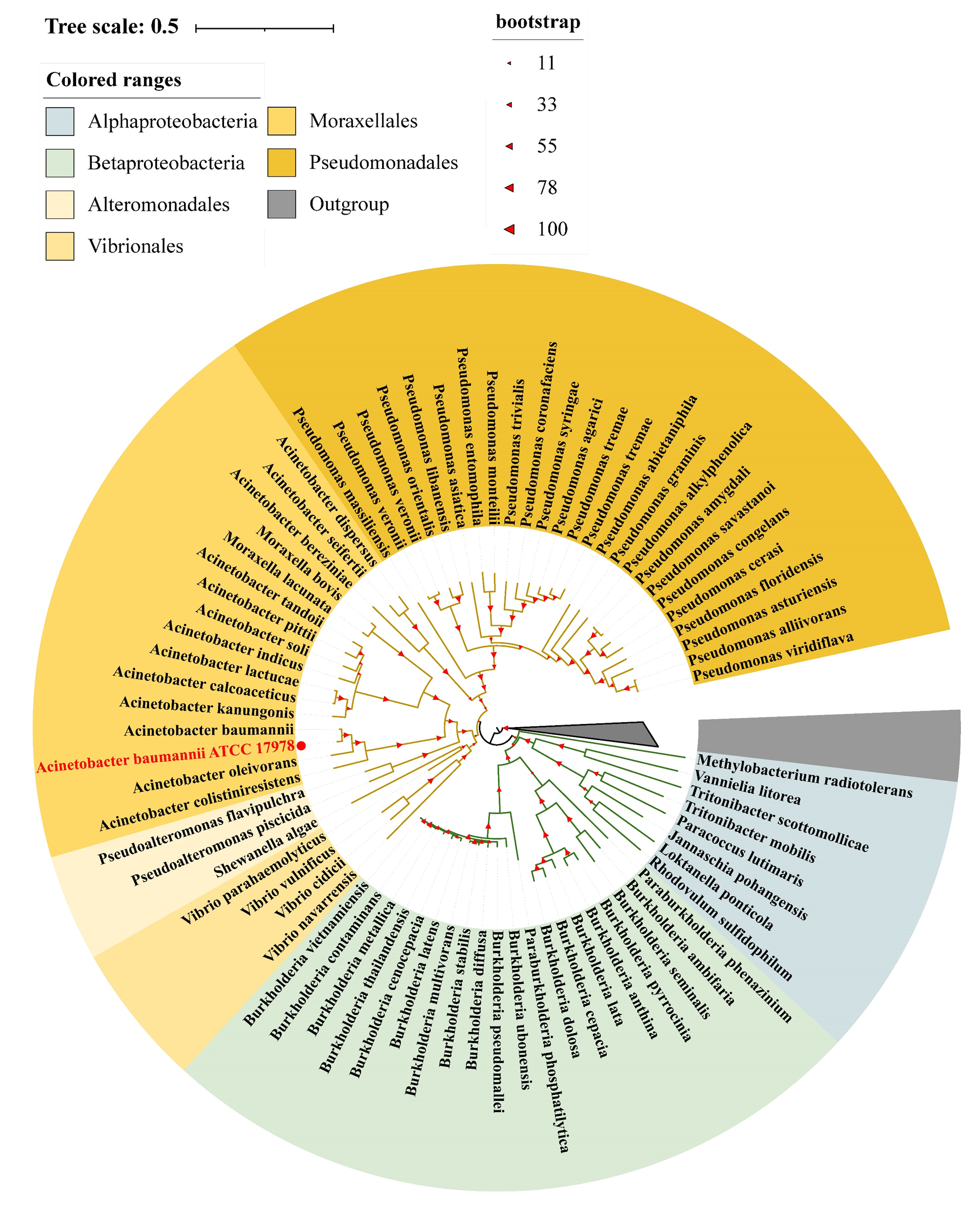

Supplement: FIG S3 [file mbio.03420-22-s0003.tif]

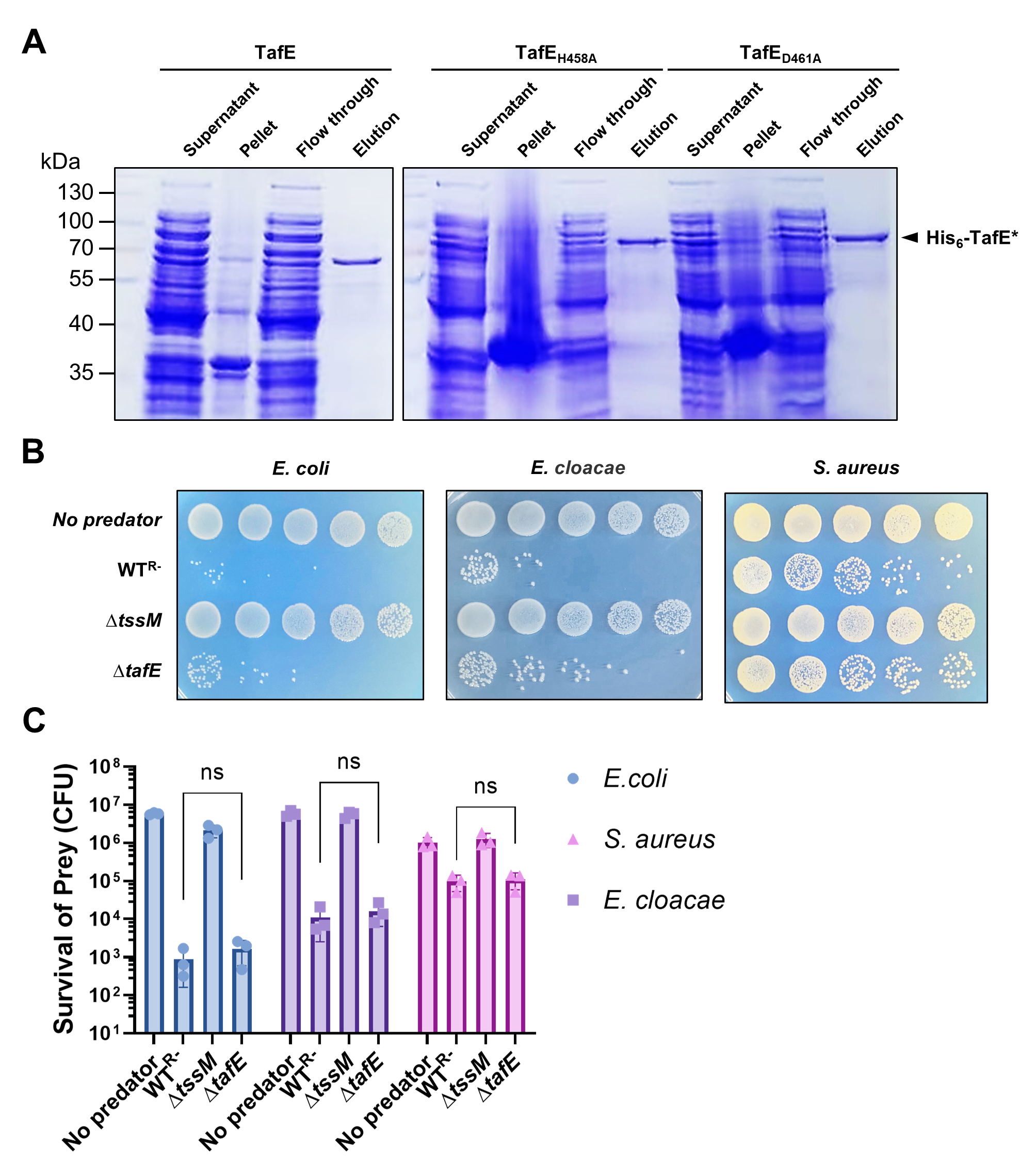

Supplement: FIG S4 [file mbio.03420-22-s0004.tif]

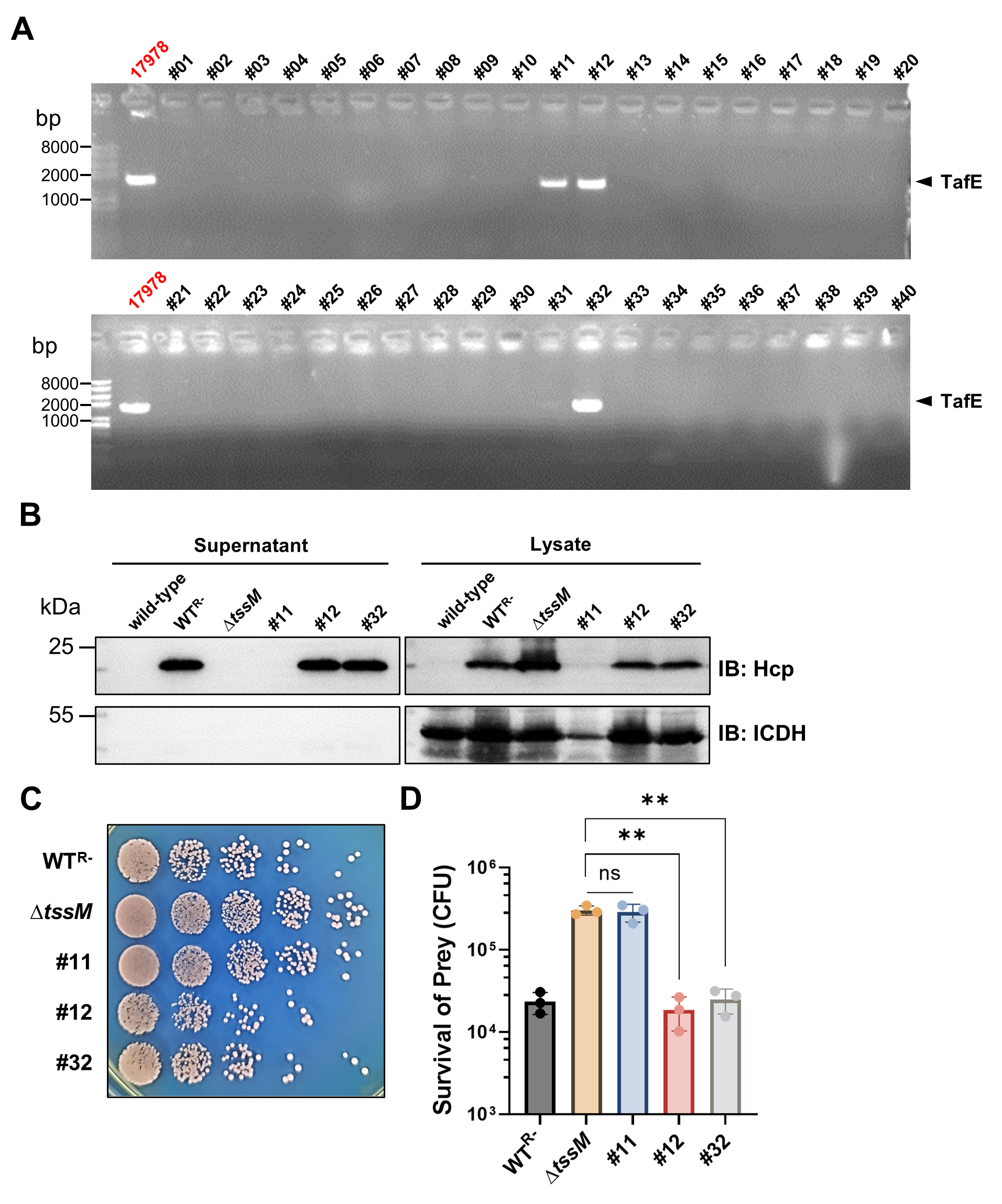

Supplement: FIG S5 [file mbio.03420-22-s0005.tif]

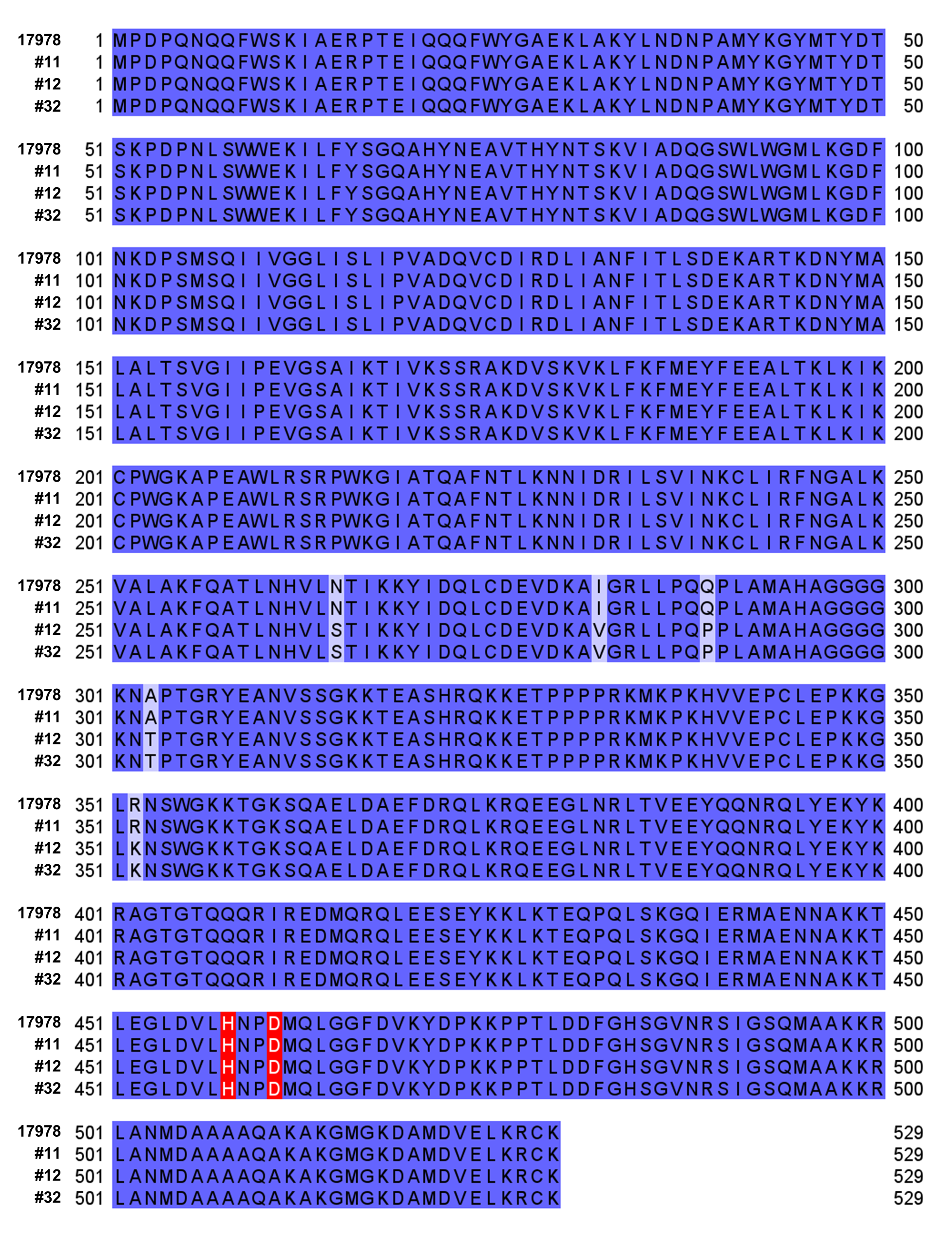

Supplement: FIG S6 [file mbio.03420-22-s0006.tif]

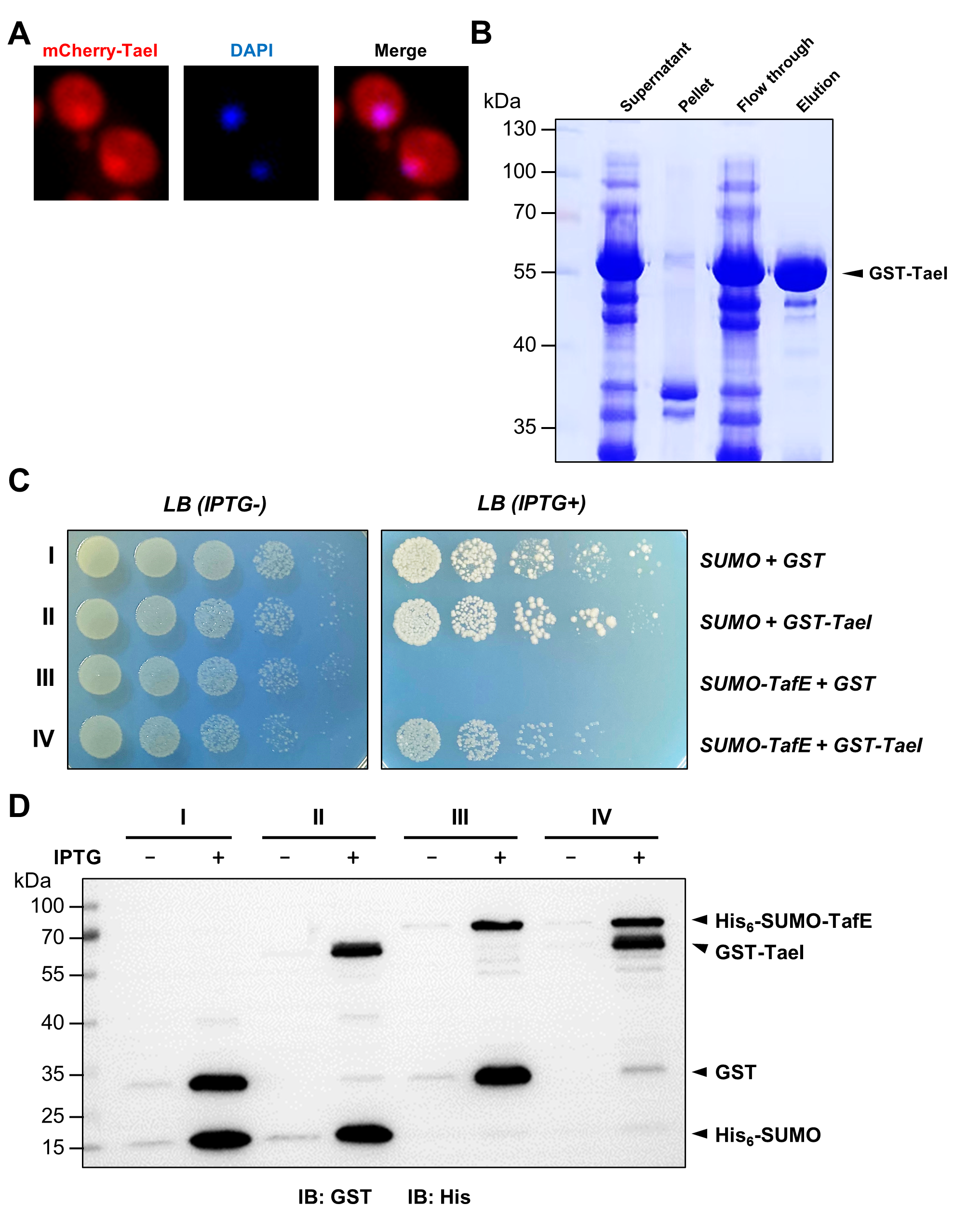

Supplement: FIG S7 [file mbio.03420-22-s0007.tif]
